# Differential responses of two fenugreek (*Trigonella foenum-graecum* L.) landraces pretreated with melatonin to prolonged drought stress and subsequent recovery

**DOI:** 10.1186/s12870-024-04835-w

**Published:** 2024-03-02

**Authors:** Masoud Maleki, Abdolali Shojaeiyan, Ali Mokhtassi-Bidgoli

**Affiliations:** 1 Department of Horticultural Science, Faculty of Agriculture, Tarbiat Modares University, Tehran, Iran; 2 Department of Agronomy, Faculty of Agriculture, Tarbiat Modares University, Tehran, Iran

**Keywords:** Drought stress, Drought recovery, Fenugreek, Saponins, Antioxidant system

## Abstract

**Background:**

Drought impairs growth, disturbs photosynthesis, and induces senescence in plants, which results in crop productivity reduction and ultimately jeopardizes human food security. The objective of this study was to determine major parameters associated with drought tolerance and recovery ability of fenugreek (*Trigonella foenum-graecum* L.), by examining differential biochemical and phenological responses and underlying enzyme activities as well as melatonin roles during drought stress and re-watering for two contrasting landraces. Moreover, the relative expression of three key genes involved in the biosynthesis pathway of diosgenin, including *SQS*, *CAS*, and *BG*, was investigated.

**Results:**

Depending on the conditions, drought stress enhanced the activity of antioxidant enzymes and the osmoregulating compounds, non-enzymatic antioxidants, hydrogen peroxide content, and lipid peroxidation levels in most cases. Severe drought stress accelerated flowering time in Shushtar landrace (SHR) but had no significant effects on Varamin (VR). Pretreatment with melatonin delayed flowering time in SHR and caused high drought resistance in this landrace. Furthermore, melatonin significantly enhanced drought adaptability in VR by improving plant recovery ability.

**Discussion:**

Based on our results plants’ responses to drought stress and melatonin pretreatment were completely landrace-specific. Drought stress caused an increase in the relative expression of *CAS* gene and ultimately the accumulation of steroidal saponins in SHR. Melatonin compensated for the decrease in biomass production due to drought stress and finally increased steroidal saponins performance in SHR. Our study showed that melatonin can improve drought stress and recovery in fenugreek, but different factors such as genotype, melatonin concentration, and plant age should be considered.

**Supplementary Information:**

The online version contains supplementary material available at 10.1186/s12870-024-04835-w.

## Introduction

Drought stress occurs when the available water for plants in the soil is low due to inadequate soil moisture at a specific time. Drought is a regular and recurrent feature of the climate that occurs in almost all areas, especially arid and semiarid regions, and aggravates with the advent of climate change [1, 2]. The changes have been such that the 2010s claimed seven of the ten warmest years on record; 2019 itself was the second-warmest year since 1851 as to both land and ocean temperatures, which resulted in the occurrence of floods, droughts, heat waves, and water scarcity. Drought events account for more than 34% of the crop and livestock production losses in the least developed and lower-middle-income countries, resulting in a substantial cost of US$37 billion for the agricultural sector [3]. Drought impairs growth, disturbs photosynthesis, and induces senescence in plants [4]. This can reduce crop productivity and ultimately jeopardizes human food security [5].

Today, extensive research is being done to produce genetically modified (GM) crops for drought resistance [6]. However, many human societies do not yet have positive opinions about using them in the future. On the other hand, some methods (e.g., conventional breeding) are very time-consuming and expensive. Alternately, plants can be prepared to face the stress through chemical treatment to more successfully tolerate biotic and abiotic stress conditions [7]. Chemical compounds have been shown to promote tolerance of various crop and non-crop species to a wide range of abiotic stresses. Phytohormones are a group of chemical signaling molecules that, in small amounts in cells, regulate responses to various external and internal stimuli and plant growth and development. Their key role in promoting plant adaptation to constant environmental changes by interfering with growth, development, source/sink change, and nutrient allocation has been well established [8, 9]. Although plant’s response to abiotic stresses depends on many factors, phytohormones are thought to be the most pivotal internal substances for regulating physiological and molecular responses during abiotic and biotic stresses [10]. Melatonin (ME), a derivative of the amino acid tryptophan, is one of the hormones found in plants that has attracted the attention of many researchers [11]. Due to the increased levels of endogenous melatonin observed after exposure of the plant to a variety of abiotic stresses, it was initially thought to be an antioxidant and plant protector in the adverse environment [11, 12]. Nevertheless, it has now been established that it plays a significant role in a variety of physiologic processes in plants, including seed germination, rooting induction, tropism, photosynthesis, growth and development, plant reproduction, fruit ripening, as well as post-harvest ripening, and fruit quality improvement [12–14].

Fenugreek (*Trigonella foenum-graecum* L.) is an annual herbaceous plant that belongs to the Fabaceae family, consumed as a spice, vegetable, and medicinal plant [15]. Fenugreek’s secondary metabolites include three main compounds: saponins, flavonoids, and alkaloids. Also, it is a good source of vitamin B1, iron, silicon, sodium, protein, amino acids, fatty acids, and dietary fiber. Furthermore, fenugreek is a rich source of soluble fiber, mucilage, galactomannan, hydroxyisoleucine, trigonelline, and saponins (such as yamogenin, tigogenin, and diosgenin) [16, 17]. Among these, diosgenin, a steroidal sapogenin, is a valuable secondary metabolite produced mostly from *Trigonella* and *Dioscorea* species and used as one of the major precursors in the synthesis of steroidal drugs [17]. So far, phytochemical, physiological, and biochemical changes in fenugreek under adverse environmental conditions, including heat [18], drought [19, 20], salt [21–23], and heavy element stresses [24, 25], have been of interest to researchers. Research has recently focused on the use of chemical agents to mitigate drought stress as well as their impact on fenugreek secondary metabolites [26–30]. However, there have been no experiments that assessed fenugreek responses to melatonin soil pretreatment under prolonged drought stress and recovery conditions. The present study aimed to test three main hypotheses. Firstly, whether the application of melatonin can have a positive effect on the drought tolerance and drought recovery of fenugreek plants under prolonged drought stress and recovery conditions. Secondly, whether the secondary metabolite content and expression of its biosynthesis pathway genes changes in fenugreek plants exposed to prolonged drought stress. Lastly, whether two fenugreek landraces may respond differently to melatonin application under drought stress and recovery conditions.

## Results

### Melatonin improves the drought resistance, recovery and adaptation

**Fig. 1:**
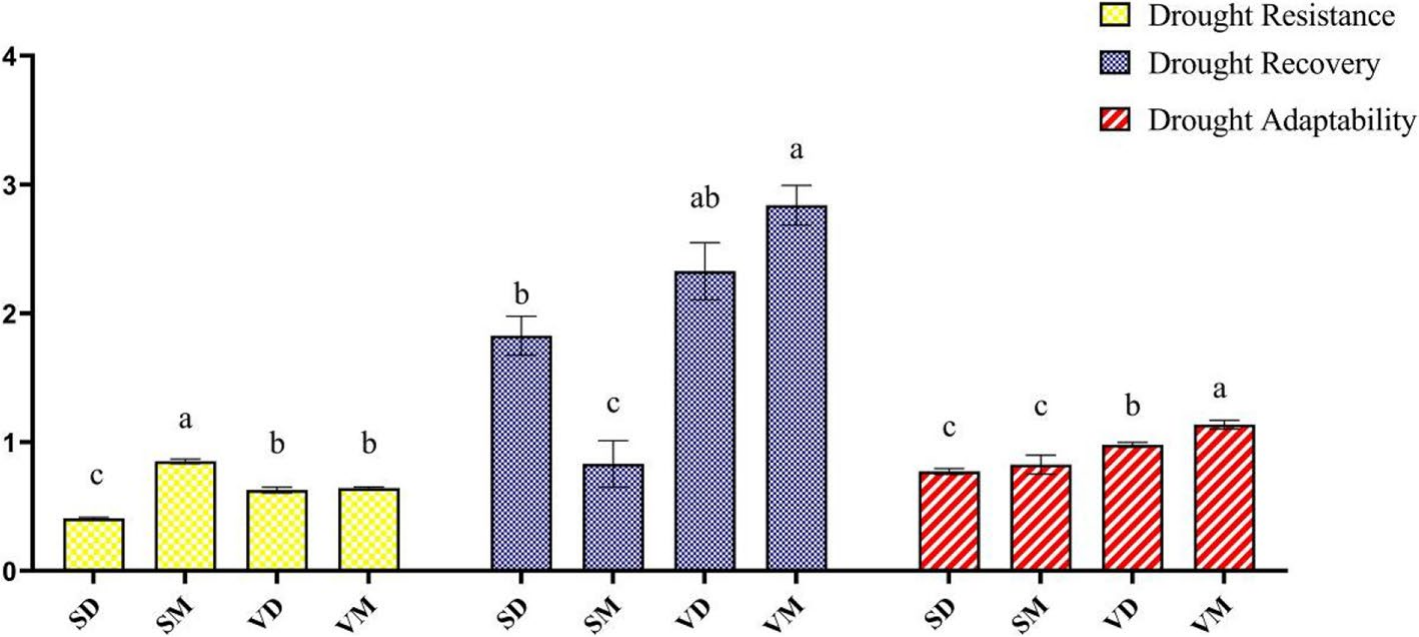
Changes in drought stress confrontation indices during drought stress and recovery in two fenugreek landraces. The bars represent the standard error of the average of three replicates. Different alphabets within the group represent significant differences among treatments according to Fisher’s test at *P* ≤ 0.05. SC: well-watered plants of Shushtar landrace; SD: Drought-stressed plants of Shushtar landrace; SM: ME-treated plants of Shushtar landrace; VC: well-watered plants of Varamin landrace; VD: Drought-stressed plants of Varamin landrace; VM: ME-treated plants of Varamin landrace

The highest resistance to drought stress was found in the ME-treated Shushtar landrace (SM) with a value of 0.8, whereas the ME-treated and control plants of Varamin (VM and VC, respectively) did not show any notable differences (Fig. 1 and Fig. S2). Drought-stressed VR plants (VD) had higher drought resistance levels than Shushtar landrace (SHR) ones. As illustrated in Fig. 1, rewatering can naturally double the dry weight of drought-stressed plants. The effect of melatonin was genotype-dependent, therefore, it reduced the recovery potential of SHR but improved that in Varamin landrace (VR). Regarding drought adaptation, the highest level was found in ME-treated VR (VM), while the lowest was observed in control and ME-treated SHR plants, with values of 0.77 and 0.82, respectively.

### Melatonin changes the enzymatic antioxidant activity during drought stress and recovery

**Fig. 2:**
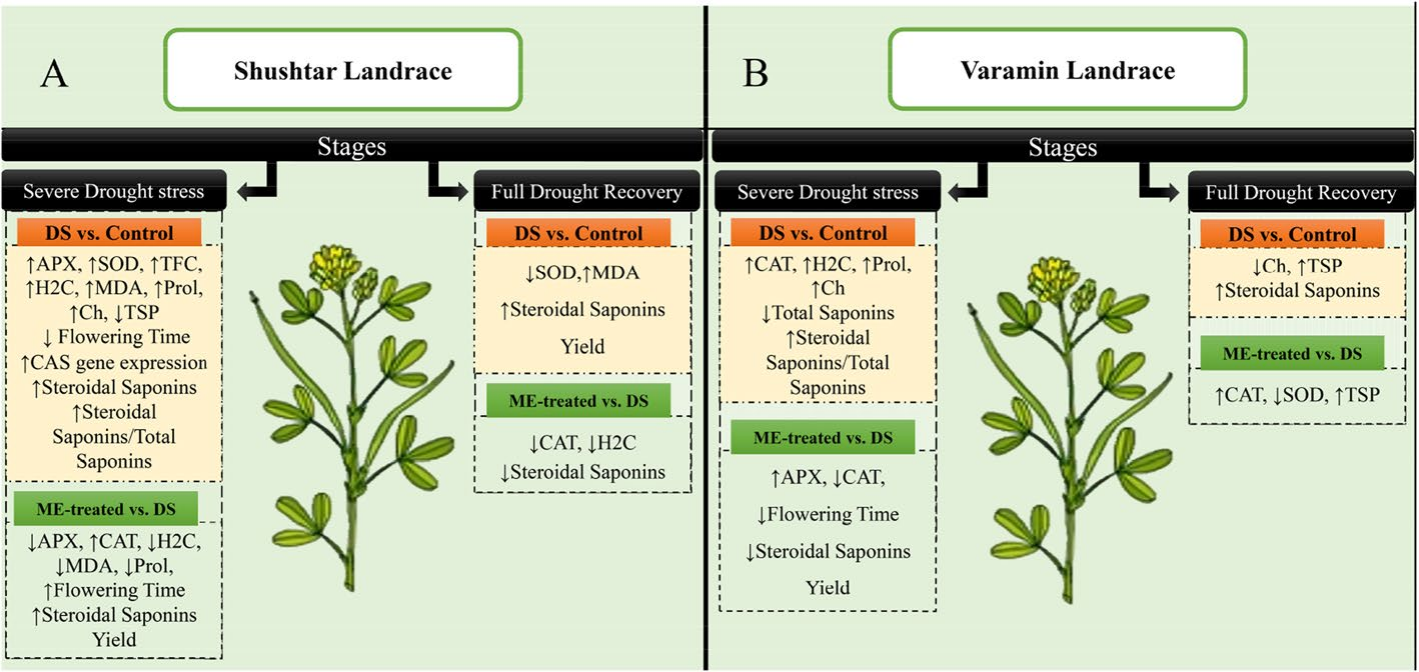
Schematic representation of the biochemical, phytochemical, and phenological responses of drought-stressed plants (DS) in comparison to controls (orange boxes) and Melatonin-treated plants in comparison to drought-stressed plants (green boxes) in two landraces of Shushtar (**A**) and Varamin (**B**) in two crucial stages of severe drought stress and full recovery. The changed parameters measured include catalase (CAT), superoxide dismutase (SOD), ascorbate peroxidase (APX), total soluble protein (TSP), total flavonoid content (TFC), H2O2 content (H2C), malondialdehyde (MDA), total carbohydrate content (Ch), and proline (Prol). The upper arrow (↑) and lower arrow (↓) denote an increase and decrease in content/activity, respectively

**Fig. 3:**
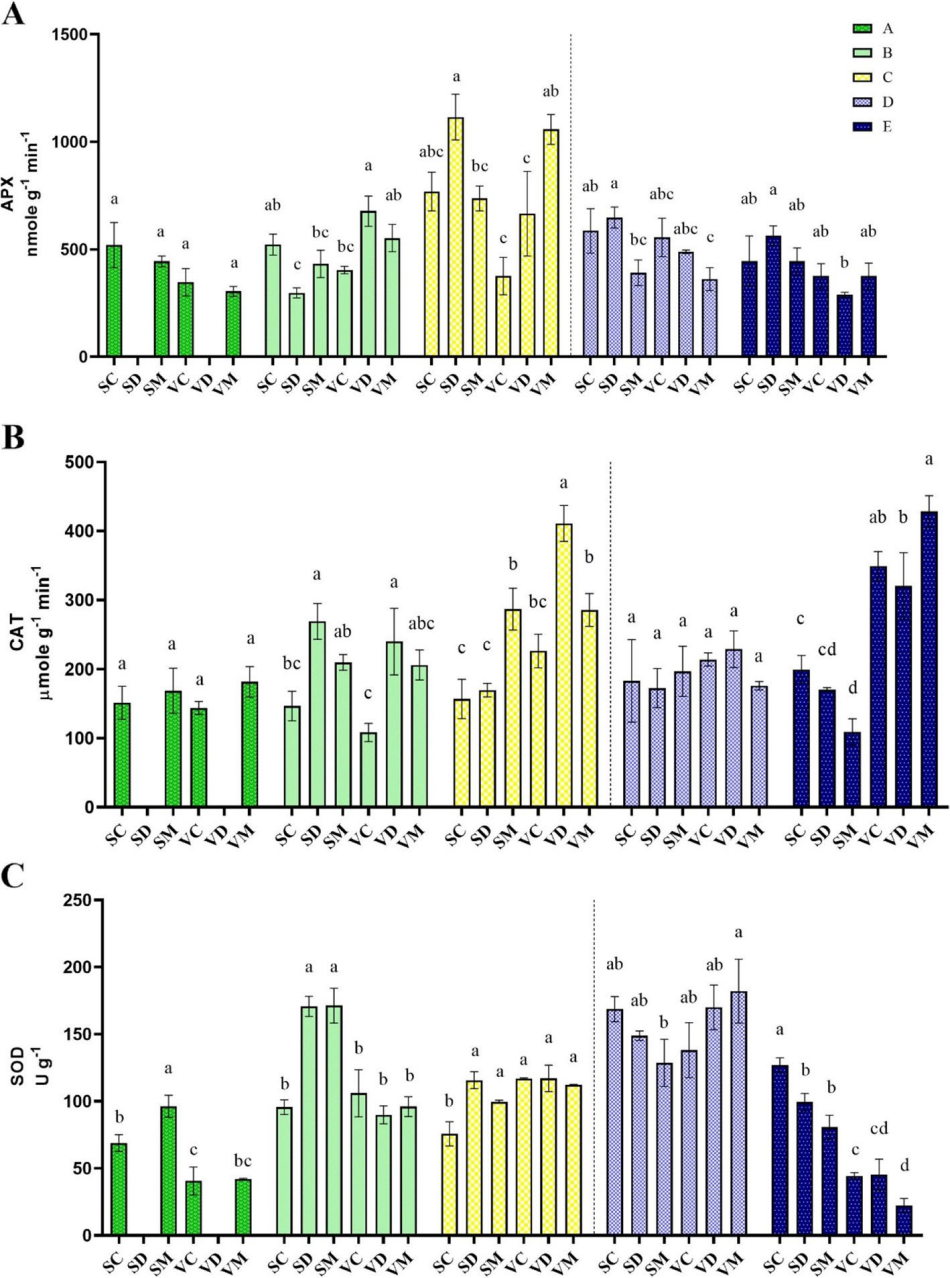
Changes in enzymatic antioxidants including APX (**A**), CAT (**B**) and SOD (**C**) during drought stress and recovery in two fenugreek landraces. The bars represent the standard error of the average of three replicates. Different alphabets within the group represent significant differences among treatments according to Fisher’s test at *P* ≤ 0.05. SC: Well-watered plants of Shushtar landrace; SD: Drought-stressed plants of Shushtar landrace; SM: ME-treated plants of Shushtar landrace; VC: well-watered plants of Varamin landrace; VD: Drought-stressed plants of Varamin landrace; VM: ME-treated plants of Varamin landrace

Drought stress significantly affects enzymatic antioxidant activity (B and C stages). At the beginning of the drought stress (stage A), regarding enzymatic antioxidant activity, significant differences were observed between the treatments except for superoxide dismutase enzyme (SOD) activity. Although the initial recovery (stage D) had no significant effects on enzymatic antioxidant activities, the CAT (catalase) and SOD activities were significantly affected by the full-recovery stage (Table 1). Drought stress increased the activity of antioxidant enzymes such as CAT, APX (ascorbate peroxidase), and SOD (Fig. 2 and 3). The activity of APX in stage A was not significantly different among the treatments. With the progression of drought stress during moderate drought stress (stage B), the activity of this enzyme showed a remarkable increase in ME-treated and drought-stressed plants of VR (VD and VM, respectively). Upon reaching the maximum drought stress (stage C), a significant growth in the activity of this enzyme was also observed in drought-stressed SHR (SD). Under the severe drought stress condition, in SHR, ME-treated plants (SM) had lower ascorbate peroxidase enzyme activity than drought-stressed plants (SD). After rewatering, the activity of this enzyme dropped significantly in drought-stressed plants, and there was no difference between treatments (Fig. 3A).

**Table 1:** Summary of variance analysis to determine whether there are statistically significant differences among the treatments (SC, SD, SM, VC, VD, and VM) regarding the measured parameters in the responses to prolonged drought stress and subsequent rewatering

	Stages				
Traits	A	B	C	D	E
CAT activity	ns	**	**	ns	***
APX activity	ns	*	*	ns	ns
SOD activity	**	***	*	ns	***
Total soluble protein	ns	***	*	ns	***
Proline	ns	ns	***	***	ns
Total carbohydrate content	ns	ns	*	**	**
Total flavonoid content	ns	ns	*	*	ns
Total phenolic compounds	ns	***	ns	*	ns
H_2_O_2_ content	**	***	*	*	*
Malondialdehyde content	ns	***	*	***	***
Total saponins content			ns		ns
Steroidal saponins content			*		ns
Steroidal saponins to total saponins ratio			*		*
Steroidal saponins yield			*		*
Total saponins yield			ns		ns

*ns* no significant. A: the early stages of growth, B: moderate drought stress, C: severe drought stress, D: initial recovery, E: full drought recovery

^*^Significant at *P* ≤ 0.05

^**^Significant at *P* ≤ 0.01

^***^Significant at *P* ≤ 0.001

In the case of CAT, SD and VD showed the highest activity during moderate drought stress (stage B). At this stage, melatonin could not cause a significant difference in ME-treated plants compared to untreated ones. During severe drought stress, the highest activity of CAT (411 μmol g^−1^ min^−1^) was observed in VD, while its level diminished in drought-stressed SHR plants (SD) to 169.6 μmol g^−1^ min^−1^. Notably, melatonin enhanced the activity of CAT in SHR (SM), whereas the opposite effect was found in VR (VM). After rewatering, CAT activity was drastically reduced in drought-stressed and ME-treated plants. But over the full-recovery period, its activity sharply increased in VR plants compared to SHR (Fig. 3B).

In the early stages of growth (stage A), intrinsically higher SOD activity was observed in SHR plants than VAR, especially in the plants pretreated with melatonin. Over time, SOD activity first increased in the leaves of drought-stressed and ME-treated SHR plants before decreasing at the next stage. Activity of this enzyme in drought-stressed and ME-treated SHR plants markedly dropped during severe drought stress. However, there were no significant differences among VR plants in terms of SOD activity during severe drought stress. Totally, the activity of SOD increased by starting recovery phase, but this increase was more evident in drought-stressed and ME-treated VR plants. Overtime, at the full-recovery stage, the SOD activity was significantly reduced in VR plants compared to SHR (Fig. 3C).

### Changes in osmoregulating compounds and non-enzymatic antioxidants contents by melatonin pretreatment

Analysis of variance showed that, in terms of the osmoregulating compounds (ORC) and non-enzymatic antioxidants (NEA) contents, there were no significant differences among the treatments during stage A. Moderate drought stress had significant effects on the total soluble protein (TSP) and total phenolic compounds (TPC) of the treatments, while it had no significant effect on the total flavonoid content (TFC), proline (Prol), and total carbohydrate (Ch) contents. All the ORC and NEA traits, except TPC, were significantly affected by severe drought stress. With the exception of TSP, rewatering had an impact on all the ORC and NEA traits in the initial recovery condition. Traits related to ORC and NEA were not affected by the full-recovery condition, except TSP and Ch (Table 1).

Prol dramatically increased in drought-stressed and ME-treated plants over the severe drought stress period, reaching a peak value of 87 nmole mg^−1^ FW in drought-stressed SHR plants (Table 2). Pretreatment with melatonin leads to a significant decline in Prol of SD plants to 62.5 nmole mg^−1^ FW when compared to VR (Fig. 2). By starting rewatering, the amount of Prol rapidly dropped in drought-stressed and ME-treated plants, but there was still a significant difference between drought-stressed plants and related controls.

**Table 2:** Changes in osmoregulating compounds and non-enzymatic antioxidants content during drought stress and recovery in two contrasting fenugreek landraces

Traits	Proline (nmol/mg Fw)					Total carbohydrate content (mg/g Fw)				
	A	B	C	D	E	A	B	C	D	E
SC	11.02 ± 1.83^a^	10.94 ± 1.51^b^	11.33 ± 0.92^c^	14.22 ± 1.21^bc^	13.91 ± 1.12^b^	22.27 ± 2.21^a^	31.11 ± 1.56^ab^	27.76 ± 1.07^d^	27.55 ± 1.78^ab^	29.4 ± 0.96^a^
SD		11.84 ± 0.08^ab^	86.99 ± 5.29^a^	44.03 ± 0.96^a^	17.07 ± 3.22^ab^		27.52 ± 1.39^b^	37.39 ± 1.85^abc^	23.89 ± 2.79^b^	27.72 ± 1.33^a^
SM	7.72 ± 0.86^a^	15.14 ± 2.96^ab^	62.52 ± 3.67^b^	19.59 ± 2.58^bc^	17.38 ± 1.85^ab^	25.11 ± 2.41^a^	31.4 ± 1.31^ab^	38.99 ± 2.01^ab^	31.14 ± 0.30^a^	28.98 ± 2.88^a^
VC	7.41 ± 1.30^a^	12.07 ± 1.84^ab^	15.1 ± 0.74^c^	14.18 ± 2.22^bc^	16.33 ± 1.85^ab^	17.56 ± 3.70^ab^	33.66 ± 1.32^a^	32.23 ± 1.62^cd^	23.31 ± 0.82^bc^	27.2 ± 0.68^a^
VD		17.72 ± 1.26^a^	51.46 ± 3.87^b^	20 ± 3.92^b^	21.97 ± 1.81^a^		28.64 ± 0.70^b^	41.66 ± 3.45^a^	23.00 ± 0.80^bc^	18.91 ± 3.20^b^
VM	8.71 ± 1.80^a^	16.05 ± 3.04^ab^	58.57 ± 4.39^b^	10.66 ± 1.62^c^	17.4 ± 0.29^ab^	10.61 ± 0.32^b^	32.17 ± 2.48^ab^	32.63 ± 2.08^bcd^	19.25 ± 1.00^c^	20.61 ± 1.32^b^
*p* Value	0.462	0.197	0.000	0.000	0.208	0.015	0.128	0.016	0.002	0.009
	Total phenolic compounds (mg Gallic acid/g Fw)					Total flavonoid Content (mg Quercetin/g Fw)				
	A	B	C	D	E	A	B	C	D	E
SC	7.66 ± 0.06^a^	12.43 ± 1.37^a^	14.74 ± 1.45^ab^	14.89 ± 0.74^a^	13.87 ± 1.06^a^	1.84 ± 0.11^a^	2.29 ± 0.11^b^	1.77 ± 0.06^c^	2.51 ± 0.09^a^	2.39 ± 0.14^a^
SD		13.02 ± 0.44^a^	15.58 ± 1.03^ab^	12.12 ± 0.23^b^	11.72 ± 0.63^a^		2.49 ± 0.10^ab^	2.72 ± 0.24^a^	2.22 ± 0.11^ab^	2.41 ± 0.17^a^
SM	6.71 ± 0.63^a^	10.00 ± 0.36^b^	16.16 ± 0.92^ab^	15.03 ± 0.67^a^	11.86 ± 0.54^a^	1.80 ± 0.02^a^	2.83 ± 0.19^a^	2.77 ± 0.15^a^	2.09 ± 0.07^b^	2.40 ± 0.12^a^
VC	7.62 ± 0.03^a^	9.57 ± 0.50^b^	14.01 ± 0.21^b^	14.01 ± 0.84^ab^	13.89 ± 1.57^a^	1.63 ± 0.15^a^	2.46 ± 0.07^b^	2.46 ± 0.14^ab^	2.47 ± 0.13^a^	2.21 ± 0.11^a^
VD		13.70 ± 0.37^a^	15.15 ± 0.97^ab^	16.04 ± 0.66^a^	13.99 ± 1.50^a^		2.61 ± 0.04^ab^	2.17 ± 0.06^bc^	2.29 ± 0.05^ab^	2.06 ± 0.13^a^
VM	6.54 ± 0.84^a^	8.10 ± 0.37^b^	17.51 ± 0.80^a^	14.20 ± 0.53^ab^	14.58 ± 1.25^a^	1.70 ± 0.03^a^	2.33 ± 0.13^b^	2.48 ± 0.11^ab^	2.00 ± 0.09^b^	2.32 ± 0.09^a^
*p* Value	0.561	0.000	0.211	0.054	0.415	0.47	0.06	0.01	0.02	0.4

SC: Well-watered plants of Shushtar landrace; SD: Drought-stressed plants of Shushtar landrace; SM: ME-treated plants of Shushtar landrace; VC: well-watered plants of Varamin landrace; VD: Drought-stressed plants of Varamin landrace; VM: ME-treated plants of Varamin landrace

The data represent the average of three replicates ± SE

Different alphabets within the group represent significant differences among treatments according to fisher’s test at *P* ≤ 0.05

Our results showed that drought stress caused a rise in Ch in the plants (Table 2). The highest Ch value was observed in drought-stressed VR plants under severe drought stress, at 41.7 mg g^−1^ FW. Unlike SHR, melatonin pretreatment considerably decreased the Ch content of drought-stressed plants in VR to 32.63 mg g^−1^ FW. After rewatering, the Ch content of the plants declined, so the lowest and highest values of that were found in the VM (19.25 mg g^−1^ FW) and SM treatments (31.14 mg g^−1^ FW), respectively. Over time, the content of Ch in SHR plants reached the same level during severe drought stress, whereas the lowest Ch content was detected in drought-stressed and ME-treated VR plants.

By comparison, TPC of drought-stressed plants of VR was improved during moderate drought stress, while a significant reduction was observed by melatonin pretreatment in both landraces (Table 2). In regard to TPC, there was no significant difference between the treatments during severe drought stress, except that melatonin significantly improved its accumulation in VR in comparison to its control. During the initial recovery stage, its level was significantly reduced only in SD, but no significant changes were observed in other treatments. While the TFC of drought-stressed and ME-treated SHR plants considerably increased during severe drought stress, its level in VR remained unchanged. In this stage, the highest and lowest values of TFC, 2.77 and 1.77 mg Quercetin g^−1^ FW, were found in SM and VM plants, respectively. After rewatering, ME-treated plants had the lowest values of TFC compared to their respective well-watered counterparts.

**Fig. 4:**
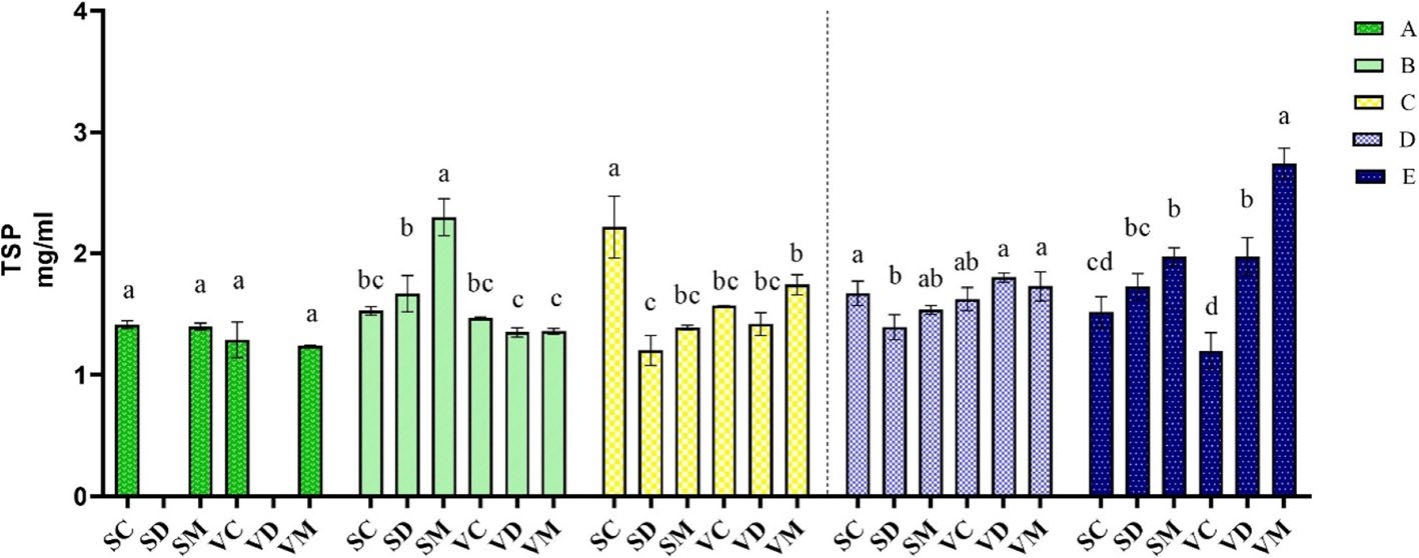
Changes in total soluble protein (TSP) during drought stress and recovery in two fenugreek landraces. The bars represent the standard error of the average of three replicates. Different alphabets within the group represent significant differences among treatments according to Fisher’s test at *P* ≤ 0.05. SC: Well-watered plants of Shushtar landrace; SD: Drought-stressed plants of Shushtar landrace; SM: ME-treated plants of Shushtar landrace; VC: well-watered plants of Varamin landrace; VD: Drought-stressed plants of Varamin landrace; VM: ME-treated plants of Varamin landrace

During moderate stress, the level of TSP significantly increased only in SM plants (2.3 mg ml^−1^), whereas no significant changes occurred in VD and VM (Fig. 4). With the intensification of drought stress, levels of TSP indicated a drastic reduction in drought-stressed and ME-treated plants of SHR (SD and SM), while the highest value of TSP (2.2 mg ml^−1^) was observed in well-watered SHR plants (SC). Melatonin pretreatment had no significant effect on the levels of TSP in severe drought stress conditions. At the full-recovery stage, the ME-treated plants had the highest values of TSP (2.74 and 1.97 mg ml^−1^ for VM and SM, respectively) compared to their respective control and drought-stressed plants. It is noteworthy that drought-stressed and ME-treated plants of VR had a higher increase in TSP during the full-recovery stage compared to the same treatments in SHR (Fig. 2).

### Effect of melatonin on reactive species and cell membrane stability

**Fig. 5:**
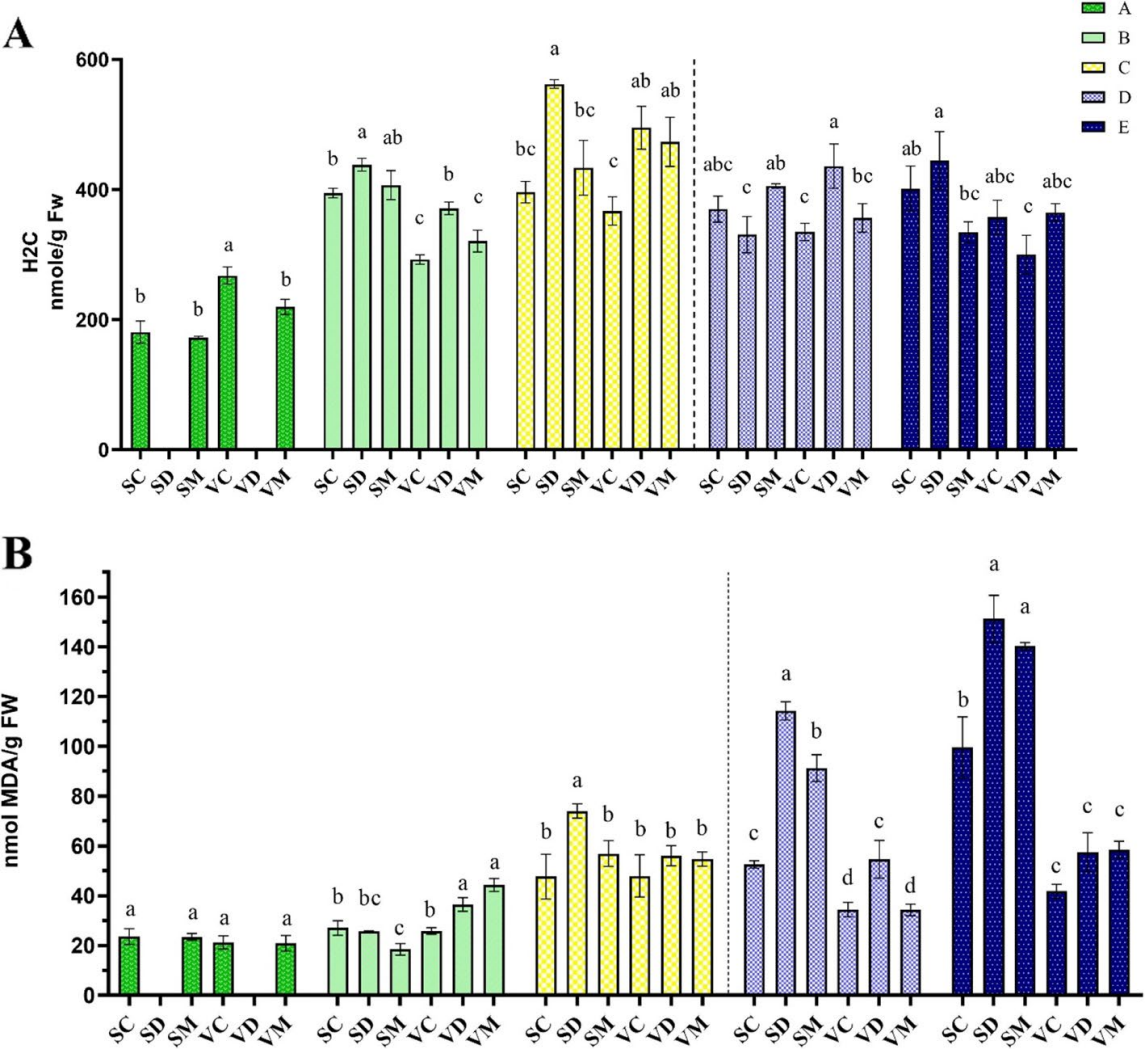
Changes in hydrogen peroxide (**A**) and lipid peroxidation (**B**) during drought stress and recovery in two fenugreek landraces. The bars represent the standard error of the average of three replicates. Different alphabets within the group represent significant differences among treatments according to Fisher’s test at *P* ≤ 0.05. SC: Well-watered plants of Shushtar landrace; SD: Drought-stressed plants of Shushtar landrace; SM: ME-treated plants of Shushtar landrace; VC: well-watered plants of Varamin landrace; VD: Drought-stressed plants of Varamin landrace; VM: ME-treated plants of Varamin landrace

Based on the analysis of variance, the prolonged drought stress and recovery can significantly affect the H_2_O_2_ content (H2C) and Malondialdehyde (MDA) content in the treatments, except for MDA during stage A (Table 1). At the early stages of growth (stage A), well-watered VR intrinsically had a higher H2C level than other treatments. The ME-treated plants showed significantly reduced levels of H2C when exposed to drought stress, while the levels of untreated plants were highly elevated during moderate drought stress (Fig. 5A). The same trend was also observed during severe drought stress, with the difference that melatonin did not have a significant effect on reducing H2C content in VR. After rewatering, the levels of H2C were remarkably reduced in drought-stressed SHR but remained unchanged in drought-stressed VR, with the highest value at 436 nmole g^−1^ FW. At the full-recovery stage (E), the levels of H2C sharply escalated in the drought-stressed SHR (SD) and reached the maximum value compared to other treatments. A relatively lesser accumulation of H2C was observed at full-recovery conditions in VR.

Moderate stress had no significant effect on the accumulation of MDA in SHR but significantly increased its levels in drought-stressed and ME-treated VR plants (VD and VM) (Fig. 5B). With the intensification of drought stress, the MDA accumulation in SD was significantly increased to 74 nmole MDA g^−1^ FW, comparatively higher than the other treatments. Melatonin pretreatment effectively diminished MDA to 56.9 nmole MDA g^−1^ FW in SM. There were no significant differences among VR treatments during severe drought stress. After rewatering, the levels of MDA in drought-stressed and ME-treated plants of SHR and drought-stressed VR were significantly higher than their respective controls. The amount of MDA in the plants pretreated with melatonin was noticeably lower when compared to the drought-stressed plants. Over time, the MDA contents were dramatically enhanced in SHR, with the highest value at 151.2 nmole MDA g^−1^ FW for SD, while the contents of MDA approximately remained steady in VR treatments.

### The Effect of melatonin on flowering time of fenugreek under severe drought stress

**Fig. 6:**
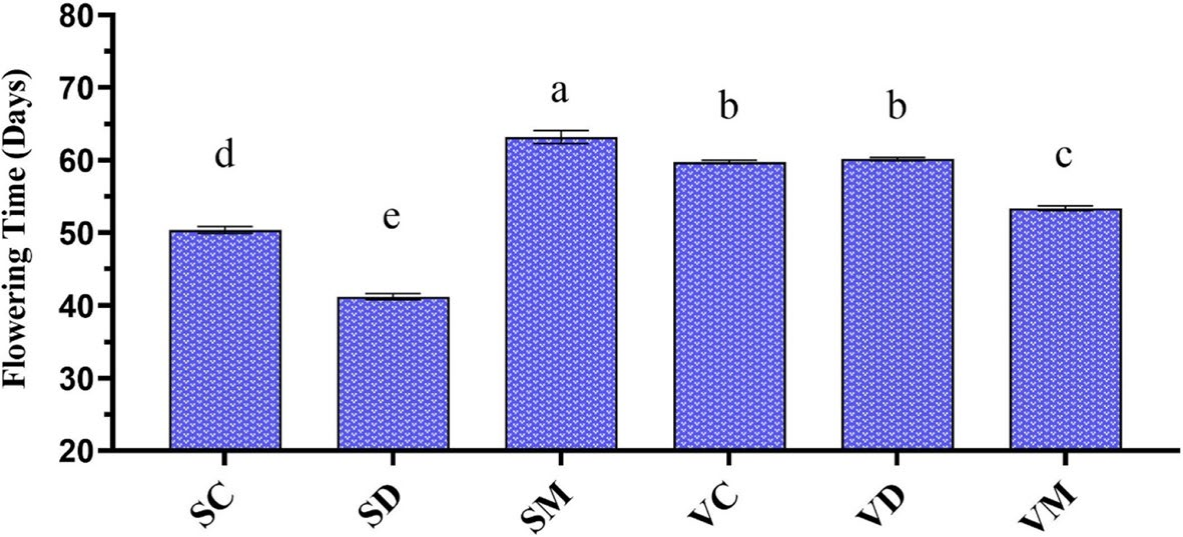
Changes in flowering time during severe drought stress in two fenugreek landraces. The bars represent the standard error of the average of four replicates. Different alphabets within the group represent significant differences among treatments according to Fisher’s test at *P* ≤ 0.05. SC: Well-watered plants of Shushtar landrace; SD: Drought-stressed plants of Shushtar landrace; SM: ME-treated plants of Shushtar landrace; VC: well-watered plants of Varamin landrace; VD: Drought-stressed plants of Varamin landrace; VM: ME-treated plants of Varamin landrace

Our results indicated that the SHR and VR landraces are intermediate-flowering (50.4 days) and late-flowering (59.8 days) plants under normal conditions, respectively (Fig. 6). With the advent of severe drought stress, flowering time was accelerated in SHR by about 9 days but had no significant effects on VR. Pretreatment with melatonin delayed flowering time in SHR by 13 days. On the contrary, it accelerated the flowering time in VR by 6.5 days.

### The Effect of melatonin pretreatment on total steroidal saponins and total saponin contents during severe drought stress and full-recovery

**Fig. 7:**
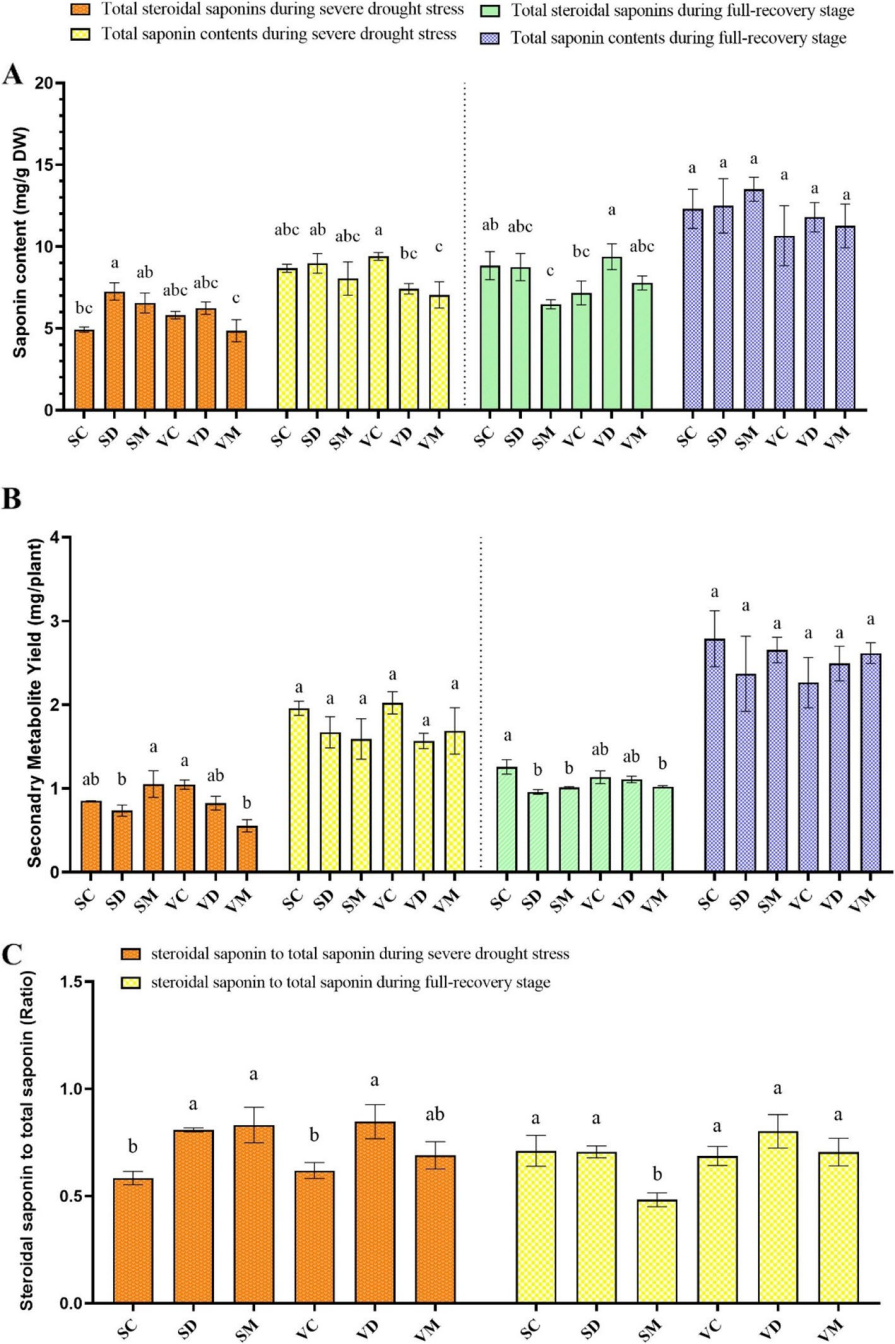
Changes in steroidal saponin and total saponin content (**A**), secondary metabolite yield (**B**), and steroidal saponin to total saponin ratio (**C**) during drought stress and recovery in contrasting fenugreek landraces. The bars represent the standard error of the average of three replicates. Different alphabets within the group represent significant differences among treatments according to Fisher’s test at *P* ≤ 0.05. SC: Well-watered plants of Shushtar landrace; SD: Drought-stressed plants of Shushtar landrace; SM: ME-treated plants of Shushtar landrace; VC: well-watered plants of Varamin landrace; VD: Drought-stressed plants of Varamin landrace; VM: ME-treated plants of Varamin landrace

Drought stress and recovery had a significant effect on the total steroidal saponins content, steroidal saponins to total saponins ratio, and steroidal saponins yield, whereas they had no significant effects on total saponins content and total saponins yield (Table 1). During severe drought stress, SD had the highest steroidal saponins at 7.25 mg g^−1^ DW, while the lowest content of 4.86 mg g^−1^ DW was found in VM. Over time, in the full-recovery stage, a remarkable increase in steroidal saponins content for nearly all the treatments was observed. At this stage, the drought-stressed VR showed a higher increase in steroidal saponins than their respective ME-treated and well-watered plants. Melatonin treatment had no significant effects on steroidal saponins during the severe drought stress and full-recovery stages (Fig. 7A).

The same levels of steroidal saponins yield were found in SM and VC (1.05 mg per plant), which were significantly higher than the other treatments during severe drought stress. By comparison, the lowest steroidal saponins yield was 0.55 mg per plant observed in VM. After rewatering, SC had significantly higher levels of steroidal saponins yield when compared to others, by up to 1.26 mg per plant (Fig. 7B).

In the case of the steroidal saponins to total saponins ratio, severe drought stress considerably enhanced steroidal saponins in drought–stressed plants when compared to the respective controls (Fig. 7C). After rewatering, there were no significant differences between the treatments except for SM, which had the lowest steroidal saponins to total saponins ratio. Melatonin pretreatment had no significant effects on steroidal saponins to total saponins ratio during severe drought stress, but it caused a lower value of steroidal saponins to total saponins ratio in SHR over full-recovery stage.

### Effect of melatonin and severe drought stress on the expression of genes involved in biosynthesis diosgenin pathway in Shushtar landrace

**Fig. 8:**
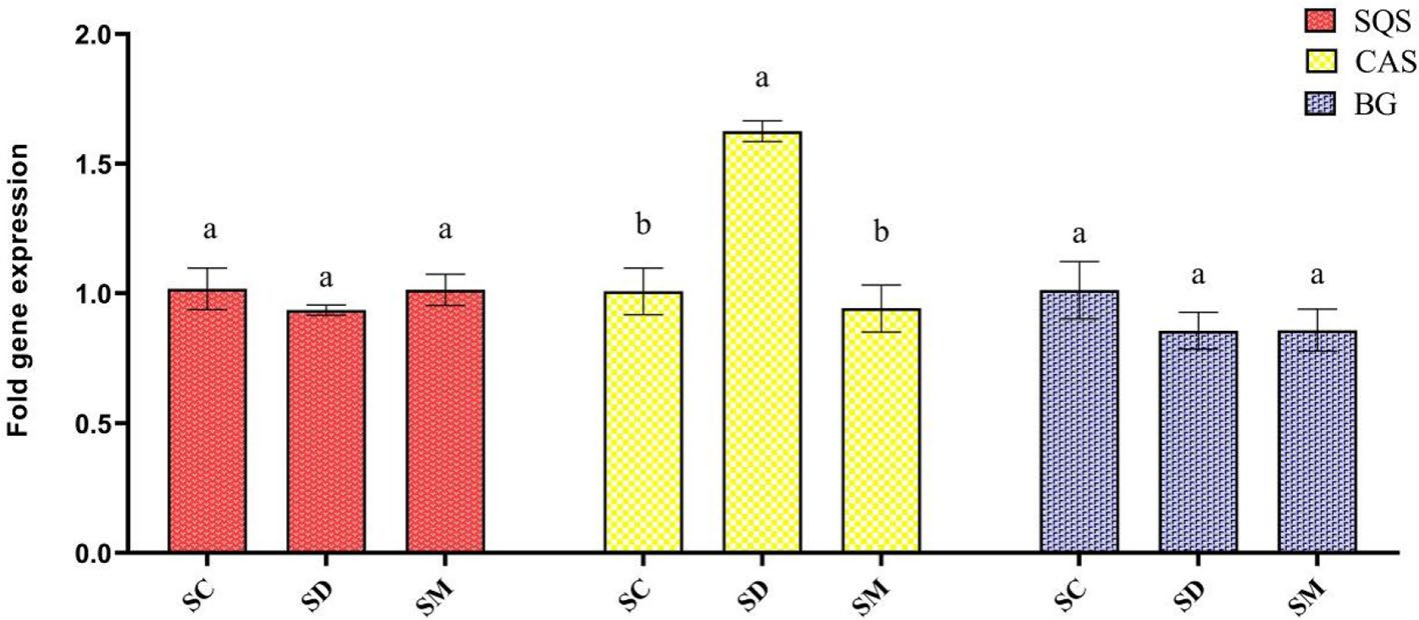
Changes in relative expression of *SQS*, *CAS* and *BG* genes during severe drought stress in contrasting fenugreek landraces. The bars represent the standard error of the average of three replicates. Different alphabets within the group represent significant differences among treatments according to Fisher’s test at *P* ≤ 0.05. SC: Well-watered plants of Shushtar landrace; SD: Drought-stressed plants of Shushtar landrace; SM: ME-treated plants of Shushtar landrace; VC: well-watered plants of Varamin landrace; VD: Drought-stressed plants of Varamin landrace; VM: ME-treated plants of Varamin landrace

To assess the effect of melatonin and severe drought stress on total steroidal saponins, a set of genes of key enzymes associated with the biosynthesis of diosgenin (i.e., one of the most dominant steroidal saponins present in the plant), viz. squalene synthase (SQS), beta-glucosidase (BG), and cycloartenol synthase (CAS), were selected for expression profiling. Severe drought stress increased the *CAS* transcript levels in drought-stressed plants by 1.63 fold in SHR. *SQS* and *BG* transcript levels did not change under severe drought stress. Our results indicated that melatonin pretreatment had no significant effects on the relative expression of genes involved in diosgenin biosynthesis in SHR under severe drought stress (Fig. 8).

## Discussion

### Melatonin pretreatment effectively impacts drought tolerance, recovery, and adaptation

In this study, we evaluated the phytochemical, biochemical, phenological, and molecular responses of two contrasting fenugreek landraces during prolonged drought stress and recovery in five stages (A-E), including early-growth (A), moderate drought stress (B), severe drought stress (C), initial-recovery (D), and full-recovery (E) stages. The Varamin landrace (VR) exhibited a higher degree of tolerance to severe drought stress compared to the Shushtar landrace (SHR) (Fig. 1). The VR landrace also displayed superior drought recovery and adaptation abilities in comparison to the SHR landrace. Melatonin pretreatment enhanced drought recovery and adaptation the VR landrace but did not affect drought resistance. In the SHR landrace melatonin pretreatment improved drought resistance but diminished drought recovery. However, our study found that melatonin pretreatment had no significant impact on drought adaptation of SHR (Fig. 1).

### Melatonin enhances drought tolerance and recovery through antioxidant enzymes activity

In the present study, melatonin pretreatment increased the activity of the APX and CAT in VR and SHR plants during severe drought stress, respectively (Fig. 2 and 3). During prolonged drought stress, the activity of antioxidant enzymes would lead to detoxifying ROS and reducing oxidative stress. Melatonin has been shown to increase the activity of antioxidant enzymes in drought-stressed plants, thus overcoming the detrimental effects of oxidative damage and ultimately protecting the plants from drought stress [31–33]. This is achieved by regulating the ascorbate–glutathione (AsA-GSH) cycle, which provides a homeostasis mechanism for direct destruction of ROS generated during drought stress [34, 35]. The regulation of enzymatic antioxidants by melatonin is due to the adjustment of the transcript levels of key genes encoding these enzymes [35, 36]. This regulation can be attributed to fluctuations in key signaling pathways such as the mitogen-activated protein kinase (MAPK) cascade pathway, especially during drought stress conditions [32]. Up-regulation of antioxidant enzymes, particularly CAT and APX, is very effective in hydrogen peroxide decomposition [37, 38], which ultimately reduces cell membrane damage. Our results showed that catalase enzyme activity plays a key role in improving drought tolerance, especially during severe drought stress. It has been reported that drought-tolerant plants of amaranth, rapeseed, sugarcane, and alfalfa demonstrate elevated CAT activity during drought stress conditions [38, 39]. Dai et al. [40] also reported that CAT is the only enzymatic antioxidant that exhibits higher activity in drought-tolerant canola during severe drought stress. Hence, the application of melatonin to the drought-sensitive landrace of SHR led to an increase in CAT activity, a decrease in APX activity, and an insignificant change in SOD activity (Fig. 2A). This enhancement improved the plant’s tolerance to drought stress, which is consistent with recent findings in alfalfa [12].

After rewatering, ME-treated plants of VR initially showed an increase in SOD activity, but over time, it declined while CAT activity enhanced (Fig. 2 and 3). SOD is the first defense enzyme that facilitates the conversion of superoxide to hydrogen peroxide, which is further scavenged by CAT activity [41]. High levels of CAT and SOD activity have been linked to an increased efficiency of ROS detoxification during the recovery process [19]. The rise in SOD activity in VR after rewatering can be attributed to the restoration of metabolic processes, leading to an increase in superoxide formation [42]. Higher SOD and APX activities during the full recovery were negatively associated with drought adaptability, while CAT activity was positively associated with drought recovery and adaptability (Table 3). It is worth mentioning that, antioxidants effectiveness is influenced by various factors such as the intensity of stress, the plant species, the developmental stage, the age of the leaf, and its position in terms of exposure to light [43].

**Table 3:** Correlations(r) between drought stress confrontation indices and measured characteristics

	Drought Stress			Recovery		
Trait	Drought	Recovery	Adaptability	Drought	Recovery	Adaptability
APX	-0.57	0.36	0.07	-0.35	-0.37	-0.59^*^
CAT	0.34	0.22	0.39	-0.27	0.90^***^	0.89^***^
SOD	-0.60	0.56	0.23	-0.23	-0.70^*^	-0.92^***^
TSP	0.31	0.50	0.79^**^	0.24	0.57^*^	0.84^***^
TFC	0.05	-0.49	-0.45	-0.07	-0.27	-0.26
H2C	-0.64^*^	0.288	-0.074	-0.58^*^	0.00	-0.33
MDA	-0.64^*^	-0.16	-0.58^*^	-0.10	-0.80^**^	-0.88^***^
TPC	0.15	0.19	0.35	0.04	0.55	0.62^*^
Ch	0.12	-0.32	-0.38	0.07	-0.67^*^	-0.66^*^
Prol	-0.51	-0.26	-0.57	0.02	0.21	0.20
DR	1.00	-0.53	0.07	1.00	-0.45	0.16
ADAP	0.07	0.80^***^	1.00	0.16	0.80^**^	1.00
FLT	0.91^***^	-0.28	0.25			

*TSP* Total soluble protein, *TFC* Total flavonoid content, *H2C* H_2_O_2_ content, *MDA* Malondialdehyde, *TPC* Total phenolic compounds, *Ch* Total carbohydrate content, *Prol* Proline, *DR* Drought resistance, *ADAP* Drought adaptation, *FLT* Flowering time

^*^Significant at *P* ≤ 0.05

^**^Significant at *P* ≤ 0.01

^***^Significant at *P* ≤ 0.001, ns: no significant

### Protecting total soluble protein levels and modulating carbohydrates improves drought adaptation

Under drought stress, osmotic adjustment plays a crucial role in maintaining cell turgor while simultaneously reducing water potential. This process enables water absorption during drought stress for many species and is associated with an increase in proline and carbohydrate contents [44, 45]. Soluble proteins, soluble sugar, and proline are essential components that contribute to the maintenance of cell turgor and water balance. Melatonin effectively decreased proline and carbohydrate levels during severe drought stress (Table 2), likely by mitigating the deleterious effects of drought stress. This finding is consistent with the recent research on *Coffea arabica* [42] and maize [46]. Zamani et al. [47] also reported that melatonin treatment at various concentrations significantly reduces the proline content in fenugreek under drought stress. In a study on Arabic coffee, Campos et al. [42] found that melatonin treatment reduces the accumulation of soluble sugars in plants' leaves while increasing them in the root tissue under drought stress. They attributed this reduction to the plant’s possible need to allocate more sucrose to the roots in response to drought stress and a deficit in the rate of photosynthesis [42].

After rewatering, a significant decline in carbohydrates and proline levels of VR was observed (Table 2). The rapid breakdown of carbohydrates and proline in plants helps them supply sufficient energy for restoration after rewatering [45, 48]. The VR landrace with higher recovery ability, showed a salient decrease in carbohydrates at full-recovery time (Fig. 2), supported by the inverse association between carbohydrates and recovery/adaptability indices (Table 3).

Under severe drought stress, the total soluble protein (TSP) content of SHR plants fell significantly in ME-treated and drought-stressed plants (Figs. 2A and 4). It has been reported in recent studies that under water scarcity, melatonin is capable of enhancing energy production by regulating the expression of glycolytic proteins and electron transport in the respiratory chain [36]. Furthermore, it has been approved that the TSP levels decrease in *Triticum aestivum* [49], *Argania spinosa* [50], and *Dendrobium moniliforme* [51] during drought stress, with an increase in TSP levels occurs upon rewatering. Under stress conditions, soluble proteins aid in the hydration of cellular membranes and protect organic molecules from oxidative damage [52]. In wheat, it has been found that the drought-sensitive variety experiences a greater decrease in soluble protein levels under severe and moderate drought stress [49], which is consistent with the findings of the current study. Additionally, Soni and Abdin [53] reported that the soluble protein levels of the *Artemisia annua* plant increased at the beginning of the stress, but decreased at the flowering stage and with the intensification of the stress. The reduction of soluble proteins under severe drought stress can be explained by three factors: firstly, their oxidation by ROS [51]; secondly, the conversion of TSP into amino acids, particularly proline, aids in lowering the osmotic potential of the cell. Thirdly, severe drought stress causes a reduction in chlorophyll content, which restricts photosynthesis and alters carbon and nitrogen metabolism [53]. In order to recover from drought stress in the absence of water and nutrient resupply, plants require a minimum capacity of amino acids that can be obtained either through protein degradation or an existing pool [54]. Interestingly, our results indicated that higher levels of TSP during severe drought stress and rewatering enhanced drought adaptability through the improvement of drought recovery, although they did not contribute to drought resistance (Table 3).

### Melatonin effectively reduces ROS and lipid peroxidation in drought-sensitive landrace

Under drought stress conditions, the stomatal closure causes a decrease in carbon assimilation and an increase in the ratio of NADPH:NADP^+^. This subsequently leads to the excessive reduction of the components within the electron-transport chain and finally the production of ROS in the chloroplast by the reaction of electrons with water. The overproduction of ROS during prolonged drought stress cannot be counterbalanced by the antioxidant system, resulting in detrimental oxidative events that ultimately cause cell death [55]. With the intensification of drought stress, the levels of H2C (a strong ROS) and MDA increased in fenugreek landraces, particularly in the drought-sensitive landraces (SHR) (Fig. 2A). However we found that melatonin can reduce ROS and lipid peroxidation in drought-stressed SHR plants during severe drought stress (Fig. 5). Previous studies have also shown that melatonin has the ability to reduce ROS and MDA levels in other plant species, such as lemon verbena [56], lime [31], maize [46], and alfalfa [12] under drought stress condition. Melatonin could easily be located on the hydrophilic side of the membrane bilayer and protects the lipid membrane by directly neutralizing reactive species, stabilizing microsomal membranes, and maintaining optimal membrane fluidity [57, 58]. Interestingly, we found that melatonin did not affect MDA levels in drought-tolerant landrace (VR) during severe drought stress (Fig. 5). The organization of melatonin in the lipid membrane depends significantly on its concentration. At low concentrations, melatonin molecules align parallel to the lipid tail, but at high concentrations, they align parallel to the lipid membrane [58]. Higher levels of melatonin have been shown to decrease the rigidity and order of the cell membrane, thereby improving fluidity and reducing lipid peroxidation [59]. The present study also revealed that lower levels of H2C and MDA during severe drought stress were associated with increased resistance to drought stress (Table 3). Additionally, during the full-recovery stage, there was a negative correlation between MDA levels and drought recovery, as well as between H2C and drought stress resistance. MDA levels also showed a negative correlation with drought stress adaptability.

### Melatonin promotes tolerance to drought stress in drought-sensitive landrace by delaying flowering time

Our results showed that severe drought stress accelerates the flowering process in SHR plants, but it had no effect on the VR landrace (Fig. 2A and 6). Previous studies have also confirmed that drought stress reduces the vegetative period and flowering time in various plant species [49, 60]. The successful transition from vegetative to reproductive growth is crucial for a plant’s evolutionary success, but this stage can be vulnerable to environmental stresses, leading to yield losses. Plants have developed the strategy of altering flowering time in response to different stress conditions, such as pathogen infection, heat, salinity, and drought to improve chances of reproduction [61]. Drought stress during the flowering and pod formation stages of fenugreek can significantly reduce yield, and the extend of this reduction is depends on the genotype [62]. Consistent with the present results, Hosseini et al. [63] also reported that drought stress can accelerate the flowering time in landraces of fenugreek in Iran. In fact, plants respond to severe drought stress by exhibiting earlier anthesis and maturity, which is a well-developed drought-escape mechanism observed in some plants [49]. Besides, plants can minimize water loss to prevent dehydration, a strategy known as “drought avoidance”. Recent research in *Arabidopsis* has suggested that floral pathways play a key role in modulating drought stress tolerance, with long-term drought stress accelerating flowering, while short-term drought stress delays it [61]. However, it is important to note that early flowering, which guarantees survival under severe drought stress, may come at the cost of reducing plant yield [64]. Therefore, it can be concluded that the early-flowering landrace, SHR, utilized the escape mechanism to cope with severe drought stress, while the later-flowering landrace, VR, employs the drought tolerance/avoidance mechanism.

Limited research has been conducted on the effect of melatonin on flowering. The current study revealed that melatonin promotes resistance to drought stress in SHR by delaying flowering time. This finding is further supported by the positive correlation between flowering time and resistance to drought stress, as reported in Table 3. A recent study on *Arabidopsis* clearly showed that melatonin delays flowering by affecting the expression of the flowering locus C (*FLC*) gene and DELLA proteins [65].

### The process of recovery plays a crucial role in facilitating the adaptation to drought-induced stress

Our study has shown that drought recovery plays a more pivotal role in drought adaptation than drought resistance (Table 3). Drought recovery is a complex process influenced by various factors. While both mechanisms benefit from different physiological processes, they both play a critical role in drought adaptation. However, our findings suggest that drought recovery is more crucial than drought resistance in adapting to drought stress. Our results highlight the significance of several factors such as CAT and SOD activity levels, MDA content, Ch, and TSP in determining the plant's ability to recover from drought stress. This finding is in agreement with earlier research by Chen et al. [66], who showed that drought adaptability is closely related to drought recovery, but not to drought resistance. This implies that while drought resistance is essential for the survival of drought-stressed plants, it alone is insufficient to ensure their long-term survival. Drought recovery is essential for repairing any damage caused by drought and restoring the plant’s physiological functions. In addition, our study underscore the importance of melatonin in drought adaptation by improving drought recovery and restoring metabolic functions. Also, we found that melatonin helps fenugreek plants modulate enzyme activity (CAT and SOD) and protein synthesis after rewatering.

### Melatonin enhances the production of secondary metabolites by promoting an increase in biomass yield

To survive in adverse environments, plants synthesize a variety of secondary metabolites. Plants need to establish a balance between primary and secondary metabolite production to achieve optimal growth under changing environmental conditions [67]. During drought stress, plants close their stomata, which leads to a significant reduction in CO_2_ absorption. Subsequently, the consumption of reducing equivalents (NADPH + H^+^) for CO_2_ fixation by the Calvin cycle declines, producing an excess supply of NADPH + H^+^. As a result, all metabolic processes lead to the production of highly reduced compounds such as isoprenoids, phenols, or alkaloids. Although it is known that drought stress often induces the production of these secondary metabolites, it can also negatively impact plant growth and biomass [68]. The present study found that severe drought stress causes an increase in steroidal saponins in SHR but has no significant effect on the steroidal saponins of VR plants (Fig. 2A). However, the application of melatonin caused a relative decrease in steroidal saponins and total saponins in both landraces during drought stress and recovery (Fig. 7). Interestingly, as the plants aged and recovered, just after their anthesis, the value of total saponins and steroidal saponins significantly increased in both landraces. This aligns with previous studies that reported increased levels of saponins and diosgenin in fenugreek during drought stress, particularly after anthesis [20, 69]. Notably, the ratio of steroidal saponins to total saponins increased in both landraces during severe drought stress. However, this finding contradicts recent studies [70–72], while aligning with a study on Quinoa [73], indicating that the observed effect may be influenced by genotype-dependent factors [29, 71].

Despite melatonin treatment leading to a decrease in steroid saponins in both landraces, increasing biomass production in SHR resulted in an improvement in its secondary metabolite production yield (Fig. 2 and 7B). It is worth mentioning that little research has been done on the effect of melatonin on the production of secondary metabolites in plants. A study on fenugreek reported trigonelline escalation in seeds when the plants were subjected to severe drought stress and melatonin treatment [47]. Moreover, some studies have shown that it can help plants overcome the adverse physiological effects of drought stress and improve the performance of essential oil production [31, 56, 74, 75].

### Drought-induced stress significantly influences diosgenin biosynthesis through the upregulation of *CAS* expression

Melatonin pretreatment did not affect the expression of studied genes in the SHR landrace. *CAS* and *SQS* genes have been introduced as key genes associated with diosgenin biosynthesis [76]. Among the studied genes (*CAS*, *SQS,* and *BG*) in this research, only *CAS* showed a relatively increased expression level, which can explain the increase of steroidal saponins content (such as diosgenin) in SHR under severe drought stress (Fig. 8). The SQS enzyme is a vital component in the process of converting two molecules of Farnesyl diphosphate into squalene. Similarly, the CAS enzyme is actively involved in the transformation of 2,3-oxidosqualene into Cycloartenol, which serves as a precursor for the final conversion into diosgenin. In the last step of diosgenin biosynthesis, β-glucosidase (*BG*) plays a crucial role by catalyzing the removal of the glucosyl residue [77].

However, the precise mechanism of diosgenin biosynthesis is still ambiguous, and requires further research. Previous studies conducted on fenugreek [77–79] and *Dioscorea zingiberensis* [76] have suggested that diosgenin may be produced from cholesterol through the intermediate cycloartenol. Consequently, it is inferred that CAS enzymes, by facilitating cycloartenol formation, play a pivotal role in diosgenin production.

Furthermore, it is worth noting that the significance of each of these genes in enhancing diosgenin content can vary depending on the specific conditions and plant species being studied. For instance, the research on licorice under controlled drought stress indicated that among the key genes involved in diosgenin biosynthesis, the relative expression of *SQS* increased, but no changes were observed in *CAS* transcript levels [80]. Therefore, further investigations are required to gain a comprehensive understanding of the precise functions of individual genes involved in the regulation of diosgenin biosynthesis.

## Conclusion

The present study was designed to investigate the biochemical, phenological, phytochemical, and molecular responses of two contrasting fenugreek landraces under the influence of melatonin pretreatment and prolonged drought stress, as well as subsequent rewatering. Melatonin action was found to be genotype-specific, with significant improvements in drought resistance and recovery response in the intermediate and late-flowering landraces, respectively. The improvement in drought resistance in the intermediate-flowering landrace was associated with an increase in CAT activity and TSP and a decline in APX activity and proline, hydrogen peroxide, and malondialdehyde contents. Melatonin promotes drought recovery in the late-flowering landrace by increasing CAT activity and the synthesis of proteins and decreasing the activity of SOD. Broadly speaking, melatonin increased drought adaptability in the late-flowering fenugreek landrace by improving plant recovery ability. It was also shown that by delaying flowering time, melatonin pretreatment in the intermediate-flowering landrace changed the drought escape to drought tolerance/avoidance mechanism, which improved drought resistance in the SHR landrace. The results of the present study showed that melatonin can improve drought stress resistance and recovery in fenugreek, but different factors such as genotype, melatonin concentration, and plant age should be considered. Drought stress caused an increase in the relative expression of the *CAS* gene and ultimately the accumulation of steroidal saponins in SHR. Melatonin compensated for the decrease in the production of secondary metabolites by increasing biomass yield in SHR. It would be interesting to assess the effects of melatonin on seed yield and the genes involved in this pathway. In order to uncover more details about melatonin's role in increasing drought adaptation, more research is needed on other plant species.

## Methods

### Plant material

Two fenugreek landraces, intermediate-flowering and late-flowering landraces, Shushtar (SHR) and Varamin (VR), respectively, were selected for the study. The experiment was performed in a greenhouse at the Agriculture Faculty of Tarbiat Modares University, Tehran, Iran. Initially, the seeds were sterilized by immersing them in 1% sodium hypochlorite for 5 min, then washed with distilled water. Afterward, the seeds were germinated in a petri dish containing two sheets of moistened filter paper in an incubator (25 ± 2̊C for 48 h). The germinated seeds were transplanted to pots (Diameter × Height: 22.5 × 20.5 cm) filled with a mixture of potting soil:cocopeat:perlite (1:1:1, by volume).

### Experimental design

A comprehensive study was conducted to ascertain the optimal concentration of melatonin (Sigma-Aldrich, CAS: 73–31-4) for plants. The study involved the testing of three different concentrations of melatonin (0, 20, and 100 μM), and two application methods (irrigation or foliar spray) at different times (once and twice). Following a thorough analysis, the study established that the most favorable outcomes were achieved by applying a concentration of 20 μM using the irrigation method twice with a one-week interval. The first treatment application was one week prior to the onset of drought stress, while the second was just before that (Fig. S1). Control pots were irrigated with distilled water in the same volume, without any melatonin application. Drought stress was imposed by withholding irrigation for 12 and 21 days on the 33-day-old plants. The soil moisture levels at which moderate and severe drought stress were induced were 36% FC and 16–18% FC, respectively. The experiment was carried out in five stages of sampling (A to E). To begin with, at the onset of drought stress, sampling was conducted for both landraces for control and ME-treated plants in optimal water conditions (stage A). The moderate drought stress stage (stage B) was reached after 12 days of the last watering of the plants. Following this, the severe drought stress stage was reached after 21 days of the last watering (stage C), and the recovery process was initiated at the end of this stage by re-watering all the drought-treated plants. After three days of re-watering, sampling was done for the initial-recovery stage (stage D). Finally, the full-recovery stage was assessed by sampling one week after re-watering (stage E). For the duration of the experiment, the control plants were consistently watered twice a week to maintain a soil moisture content of 70–100% FC in their pots. At each stage of the experiment, the collected samples were promptly transferred to the freezer at -80̊C by means of a liquid nitrogen tank for storage and future use. In the present study, the soil moisture contents at stages B and C were evaluated according to the method outlined by Chakma et al. [81].

### Enzymatic antioxidants assay

First, 60 mg of ground-frozen leaf samples were homogenized in 600 μL of extraction buffer containing 100 mM sodium phosphate buffer (pH 7.8), 0.1 mM ethylenediaminetetraacetic acid (EDTA), 1% w/v polyvinyl pyrrolidone (PVP), and 0.5% v/v triton X-100 under cold conditions [82]. Then, the homogenate was centrifuged at 12,000 g for 10 min at 4̊C to acquire the supernatant. The supernatant was used as an enzyme extract to measure protein and antioxidant enzymes. Total soluble protein content was measured using bovine serum albumin (BSA) as a standard, according to Grintzalis et al. [83]. Catalase (CAT) activity was measured according to the method explained by Tiwari et al. [82], with some modifications. A decrease in the absorbance of H_2_O_2_ was measured for 1 min at 240 nm in a microplate reader. One unit of CAT activity is defined as the amount of required enzyme to degrade 1 μmol of H_2_O_2_ per minute. Ascorbate peroxidase (APX) activity was measured based on Murshed et al. [84] with some modifications by recording the absorbance at 290 nm. A unit of APX activity was defined as the amount of enzyme that is necessary to oxidize 1 μmol of ascorbic acid per minute. Superoxide dismutase (SOD) activity was determined according to Sales et al. [85] by measuring the absorbance at 540 nm. Enzymes activity were measured in three technical replicates.

### Non-enzymatic antioxidants assay and osmoregulating compounds

Total carbohydrate content (Ch) and proline content (Prol) were estimated by using a hot-ethanolic extract. To prepare the hot-ethanolic extract, 40 mg of ground-frozen leaf tissue was mixed with 0.5 ml of 80% (v/v) ethanol, followed by heating at 80̊C for 20 min in a Bain-Marie. The extraction was repeated four times, and the supernatants were pooled into the same microtubes before storage at -20̊C [86].

### Total carbohydrate content

Carbohydrate content was quantified in a microplate format by the phenol–sulfuric acid method suggested by Masuko et al. [87]. The concentrated sulfuric acid (150 μL) and 5% phenol (30 μL) were quickly added to 50 μL of ethanolic extract in a microtube. Microtubes were incubated at 90̊C for 5 min in a thermoblock and cooled to room temperature for 5 min, followed by reading the absorbance at 490 nm.

### Proline content

Quantification of the proline content was performed by mixing 200 μL of the ethanolic extract with 400 μL of reaction mix, which includes 1% (w/v) ninhydrin, 20% (v/v) ethanol, and 60% (v/v) glacial acetic acid, in a microtube. Afterward, the samples were heated at 95 °C for 20 min in a Bain-Marie. After cooling to room temperature, the absorbance was measured at 520 nm using a microplate reader. The amount of proline was calculated based on a standard curve [88, 89].

### Total phenolic compounds (TPC)

TPC was measured following the method of Ainsworth and Gillespie [90] with some modifications. Briefly, 100 μL of the methanolic extract of leaf samples were homogenized with 200 μL of F–C reagent 10% (v/v) and 800 μL of Na_2_CO_3_ 700 mM in a microtube and incubated at room temperature for 2 h. The absorbance of the reaction mixture was recorded at 765 nm. TPC was estimated from a standard curve prepared with gallic acid.

### Total flavonoid content (TFC)

Total flavonoid content was determined by an aluminum chloride colorimetric assay adapted from Sembiring et al. [91]. First, 50 μL of the ethanolic extract was added to 10 μL of 10% aluminum chloride solution and 150 μL of 96% ethanol. Then, 10 μL of 1 M sodium acetate was added to the mixture in a 96-well plate. All reagents were mixed and incubated at room temperature for 40 min away from light. The absorbance was measured at 415 nm with a microplate reader (Microplate Spectrophotometer, BioTek, Epoch). The absorbance values of the samples were compared against a standard curve of known concentrations of quercetin.

### Reactive species and cell membrane stability

The H_2_O_2_ content (H2C) of the samples was determined according to Junglee et al. [92]. In brief, 25 mg of ground-frozen leaf tissue was homogenized in 1 ml of a solution containing 0.25 ml of trichloroacetic acid (TCA) (0.1% (w:v)), 0.5 ml of KI (1 M), and 0.25 ml of potassium phosphate buffer (10 mM) at 4̊C for 10 min. The homogenate was centrifuged at 12,000 g and 4̊C for 20 min, followed by transferring 200 μL of supernatant to a UV-microplate well and incubating at room temperature (20̊C-22̊C) and keeping away from light for 20 min. The absorption of the supernatants was measured at 350 nm. The H_2_O_2_ content was estimated based on the generated standard curve.

To estimate the lipid peroxidation, the MDA content of the samples was measured via the 2-thiobarbituric acid (TBA) reaction, according to the modified protocol of Hodges et al. [93]. Ground-frozen leaf samples (40 mg) were homogenized in 0.5 ml of 80% cold ethanol and centrifuged at 15,000 g for 10 min at 4̊C. The supernatants were added into 2 ml microtubes containing 1 ml of reaction reagent (20% trichloroacetic acid and 0.5% of TBA). The mixture was heated in a thermoblock (KIAGEN, Iran) at 80̊C for 30 min, then cooled on an ice bath for 5 min, and finally centrifuged at 13,500 rpm. The absorbance of 200 μL of the mixture was measured at 532 nm.

### Indices of drought stress confrontation

For calculation of drought stress confrontation indices, dry weight (DW) of the treatments were measured in 0 (D0), 21 (D21) and 28 (D28) days after drought stress implementation.

Drought resistance was calculated based on relative growth during drought stress.

$$\mathrm{Drought}\;\mathrm{resistance}\hspace{0.17em}=\hspace{0.17em}\frac{{\mathrm{Dw}}_{\mathrm{TreatmentD}\ 21}-{\text{DW}}_{\mathrm{TreatmentD}\ 0}}{{\text{DW}}_{\mathrm{ControlD}\ 21}-{\mathrm{DW}}_{\mathrm{ControlD}\ 0}}$$

Drought recovery was estimated based on relative growth during drought recovery.

$$\mathrm{Drought}\;\mathrm{recovery}\hspace{0.17em}=\hspace{0.17em}\frac{{\text{DW}}_{\mathrm{TreatmentD}\ 28}-{\text{Dw}}_{\mathrm{TreatmentD}\ 21}}{{\text{DW}}_{\mathrm{ControlD}\ 28}-{\text{DW}}_{\mathrm{ControlD}\ 21}}$$

Drought adaptability was calculated based on relative growth during the whole drought stress and recovery stages.

$$\mathrm{Drought}\;\mathrm{adaptability}\hspace{0.17em}=\hspace{0.17em}\frac{{\text{DW}}_{\mathrm{TreatmentD}\ 28}-{\text{DW}}_{\mathrm{TreatmentD}\ 0}}{{\text{DW}}_{\mathrm{ControlD}\ 28}-{\text{DW}}_{\mathrm{ControlD}\ 0}}$$

### Flowering time assessment

Flowering time was recorded as the days from sowing to when the first flower opened on half of the landrace plants.

### Preparation of extracts

The preparation of saponin-rich extract was done according to the detailed methods of Del Hierro et al. [94] and Herrera et al. [95], with some modifications. In brief, 100 mg of dried leaf samples were ground in a Retsch mixer mill (MM 400, Germany) at 30 Hz for 3 min. Samples were extracted with pure methanol at a ratio of sample to solvent of 1:10 (w/v), then vortexed and subsequently sonicated for 15 min. The extraction temperature was kept under 45̊C. The mixture was then centrifuged at 3400 g for 3 min. The collected supernatants were defatted by the addition of the same volume of hexane and centrifuged at 3400 g for 10 min. Then, the supernatant was discarded, and the methanolic bottom phase was evaporated under a vacuum at 45̊C. The pigments were removed by adding an equal volume of water and chloroform to the dried sample. The mixture was then centrifuged at 3400 g for 5 min. Afterward, the chloroform phase was removed, and the remaining saponin-crude extract was concentrated to saponin-rich extract by water-saturated n-butanol. This mixture was centrifuged at 3400 g for 5 min, and the supernatant of n-butanol was collected. This last procedure was repeated three times. All the collected supernatants were evaporated under a vacuum at 45̊C. The resulting saponin-rich extracts were kept at 4̊C until further use.

### Total saponin content quantification

Briefly, the dried methanolic extracts previously obtained were prepared at 10 mg mL^−1^ in methanol. Aliquots of 125 μL were transferred to vials, followed by adding 125 μL of freshly prepared vanillin in ethanol (8%, w/v) and 1.25 mL of sulfuric acid in water (72%, v/v). A control sample was also prepared using methanol. Samples were vortexed and heated at 60°C for 10 min. Vials were cooled in ice for 5 min, and absorbance was measured at 544 nm using a microplate reader (Microplate Spectrophotometer, BioTek, Epoch) against the control sample containing methanol. The total saponin content was calculated from a standard curve of diosgenin ranging from 100 to 500 ppm. The results were expressed in mg/g of dry weight.

### Total steroidal saponins quantification

Initially, crude saponin extracts were dissolved in pure methanol at 0.1 mg/ml. Aliquots of 150 μL were transferred to vials and dried under vacuum at 45̊C. Afterward, 1 ml of ethyl acetate was added, followed by 0.5 ml of anisaldehyde–ethyl acetate reagent (0.5:95.5, v/v) and 1 ml of sulfuric acid–ethyl acetate reagent (50:50, v/v). The mixture was gently vortexed and incubated in a Bain-Marie at 60̊C for 20 min. After cooling for 10 min in a water bath at room temperature, the absorbance was measured at 430 nm using a UV–vis spectrophotometer (Bio-Rad, SmartSpec 3000) against a blank that contained ethyl acetate in lieu of the sample.

### RNA isolation, cDNA synthesis, and gene expression analysis

In this study, the transcript abundance of three key genes of the diosgenin pathway, including squalene synthase (*SQS*), cycloartenol synthase (*CAS*), and 26-O-beta glycosidase (*BG*), was evaluated. The leaves of the landrace that produced the highest amounts of steroidal saponins during severe drought stress were applied to total RNA extraction by using RNX-plus solution (Sinaclon, Tehran, Iran). RNA integrity was assessed spectrophotometrically and by gel electrophoresis. For real-time PCR analyses, 2 μg of total RNA was converted into cDNA using M-MuLV reverse transcriptase based on the manufacturer’s protocol (Sinacolon, Tehran, Iran). Thereafter, real-time PCR was carried out using Rotor-Gene Q (Qiagene, USA) in a final volume of 20 μL containing 2 μL of cDNA (250 ng μL^−1^), 5 μmol L^−1^ of each primer, and 10 μL of master mix (RealQ Plus Master Mix Green, Ampliqon, USA). The primer sequences utilized in this study were obtained from Mohammadi et al. [77] are listed in Table S1. The thermocycler conditions were as follows: initial denaturation at 95̊C for 5 min, followed by 40 cycles of amplification (95̊C for 30 s, annealing temperature 61̊C for 30 s, and 72̊C for 30 s), and a final elongation stage at 72̊C for 5 min. A melting curve run was carried out by ramping up the temperature (0.5̊C s^−1^) between 65̊C and 95̊C, as the amplification process was completed. The *GAPDH* gene was selected as a housekeeping reference gene for qRT‐PCR. Relative quantification of gene expression was performed based on the Livak method (2^−ΔΔCt^) in the Microsoft Excel software (Microsoft Office 2016).

### Statistical analysis

The experiment was conducted according to a randomized complete design, separately for each stage, with three independent biological replicates. Gene expression analyses were performed in three technical replicates for each sample. The samples were obtained by pooling three biological replicates (equal concentrations of each RNA's replication). Flowering data for each treatment was recorded from at least 18 pots (ca. 90 plants). Statistical analysis was performed using one-way analysis of variance (ANOVA), and mean comparisons were applied using Fisher's test at *P* ≤ 0.05. Statistical analyses were performed by Minitab 17 software. Graphs were generated using GraphPad Prism 8 software.

### Supplementary Information

**Supplementary Material 1.**

## Data Availability

The datasets supporting the conclusions of this article are included within the article and its supplementary file.

## Abbreviations

ME: Melatonin
SHR: Shushtar
VR: Varamin
FC: Field Capacity
CAT: Catalase enzyme
APX: Ascorbate peroxidase enzyme
SOD: Superoxide dismutase enzyme
Ch: Total carbohydrate content
Prol: Proline content
TPC: Total phenolic compounds
TFC: Total flavonoid content
H2C: H_2_O_2_ content
MDA: Malondialdehyde
FLT: Flowering time
*SQS*: Squalene synthase gene
*CAS*: Cycloartenol synthase gene
*BG*: 26-O-beta glycosidase gene
ORC: Osmoregulating compounds
NEA: Non-enzymatic antioxidants

## References

[1] Hossain MA, Wani SH, Bhattacharjee S, Burritt DJ, Tran L-SP (2016). Drought stress tolerance in plants. Springer.

[2] Kim T-W, Jehanzaib M (2020). Drought risk analysis, forecasting and assessment under climate change. Water.

[3] Food OA (2021). The impact of disasters and crises on Agriculture and Food Security: 2021.

[4] Pal S, Zhao J, Khan A, Yadav NS, Batushansky A, Barak S, Rewald B, Fait A, Lazarovitch N, Rachmilevitch S (2016). Paclobutrazol induces tolerance in tomato to deficit irrigation through diversified effects on plant morphology, physiology and metabolism. Sci Rep.

[5] Hasanuzzaman M, Shabala L, Brodribb TJ, Zhou M, Shabala S (2019). Understanding physiological and morphological traits contributing to drought tolerance in barley. J Agron Crop Sci.

[6] Lawlor DW (2013). Genetic engineering to improve plant performance under drought: physiological evaluation of achievements, limitations, and possibilities. J Exp Bot.

[7] Savvides A, Ali S, Tester M, Fotopoulos V (2016). Chemical priming of plants against multiple abiotic stresses: mission possible?. Trends Plant Sci.

[8] Fahad S, Hussain S, Bano A, Saud S, Hassan S, Shan D, Khan FA, Khan F, Chen Y, Wu C (2015). Potential role of phytohormones and plant growth-promoting rhizobacteria in abiotic stresses: consequences for changing environment. Environ Exp Bot.

[9] Chen Z, Wang Z, Yang Y, Li M, Xu B (2018). Abscisic acid and brassinolide combined application synergistically enhances drought tolerance and photosynthesis of tall fescue under water stress. Sci Hortic.

[10] Ullah A, Manghwar H, Shaban M, Khan AH, Akbar A, Ali U, Ali E, Fahad S (2018). Phytohormones enhanced drought tolerance in plants: a coping strategy. Environ Exp Bot.

[11] Tan D-X, Reiter RJ (2020). An evolutionary view of melatonin synthesis and metabolism related to its biological functions in plants. J Exp Bot.

[12] Antoniou C, Chatzimichail G, Xenofontos R, Pavlou JJ, Panagiotou E, Christou A, Fotopoulos V (2017). Melatonin systemically ameliorates drought stress-induced damage in *Medicago sativa* plants by modulating nitro‐oxidative homeostasis and proline metabolism. J Pineal Res.

[13] Arnao MB, Hernández-Ruiz J (2019). Melatonin: a new plant hormone and/or a plant master regulator?. Trends Plant Sci.

[14] Tiwari RK, Lal MK, Kumar R, Chourasia KN, Naga KC, Kumar D, Das SK, Zinta G (2021). Mechanistic insights on melatonin-mediated drought stress mitigation in plants. Physiol Plant.

[15] Amiriyan M, Shojaeiyan A, Yadollahi A, Maleki M, Bahari Z (2019). Genetic diversity analysis and population structure of some Iranian fenugreek (*Trigonella foenum-graecum* L.) landraces using SRAP markers. Mol Biol Res Commun.

[16] Chaudhary S, Chaudhary PS, Chikara SK, Sharma MC, Iriti M (2018). Review on fenugreek (*Trigonella foenum-graecum* L.) and its important secondary metabolite diosgenin. Not Bot Horti Agrobot.

[17] Shahrajabian MH, Sun W, Shen H, Cheng Q (2021). A mini-review of Galactomannas and Diosgenin in Fenugreek. Phcog Commn.

[18] Sheikhi S, Ebrahimi A, Heidari P, Amerian MR, Rashidi-Monfared S, Alipour H (2023). Exogenous 24-epibrassinolide ameliorates tolerance to high-temperature by adjusting the biosynthesis of pigments, enzymatic, non-enzymatic antioxidants, and diosgenin content in fenugreek. Sci Rep.

[19] Maleki M, Shojaeiyan A, Mokhtassi-Bidgoli A (2021). Genotypic variation in biochemical and physiological responses of fenugreek (*Trigonella foenum-graecum* L.) landraces to prolonged drought stress and subsequent rewatering. Sci Hortic.

[20] Saxena S, Kakani R, Sharma L, Agarwal D, John S, Sharma Y (2017). Genetic variation in seed quality and fatty acid composition of fenugreek (*Trigonella foenum-graecum* L.) genotypes grown under limited moisture conditions. Acta Physiol Plant.

[21] Mickky BM, Abbas MA, Sameh NM (2019). Morpho- physiological status of fenugreek seedlings under NaCl stress. J King Saud Univ Sci.

[22] Sharma K, Sharma S, Vaishnav A, Jain R, Singh D, Singh HB, Goel A, Singh S (2022). Salt-tolerant PGPR strain *Priestia Endophytica* SK1 promotes fenugreek growth under salt stress by inducing nitrogen assimilation and secondary metabolites. J Appl Microbiol.

[23] Dadresan M, Luthe DS, Reddivari L, Chaichi MR, Yazdani D (2015). Effect of salinity stress and surfactant treatment on physiological traits and nutrient absorption of fenugreek plant. Commun Soil Sci Plant Anal.

[24] Alaraidh I, Alsahli A, Razik EA (2018). Alteration of antioxidant gene expression in response to heavy metal stress in *Trigonella foenum-graecum* L. S Afr J Bot.

[25] El Rasafi T, Bouda S, Hamdali H, Haddioui A (2021). Seed germination and early seedling growth of fenugreek (*Trigonella Foenum-Gracium* L.) under Cu, Ni and as stress. Acta Ecol Sin.

[26] Baghbani-Arani A, Modarres-Sanavy SAM, Mashhadi-Akbar-Boojar M, Mokhtassi-Bidgoli A (2017). Towards improving the agronomic performance, chlorophyll fluorescence parameters and pigments in fenugreek using zeolite and vermicompost under deficit water stress. Ind Crops Prod.

[27] Bitarafan Z, Liu F, Andreasen C (2020). The effect of different biochars on the growth and water use efficiency of fenugreek (*Trigonella foenum-graecum* L). J Agron Crop Sci.

[28] Irankhah S, Sillo F, Nerva L, Ganjeali A, Balestrini R, Chitarra W (2020). Combined effects of water deficit, exogenous ethylene application and root symbioses on *trigonelline* and ABA accumulation in fenugreek. Appl Sci.

[29] Irankhah S, Chitarra W, Nerva L, Antoniou C, Lumini E, Volpe V, Ganjeali A, Cheniany M, Mashreghi M, Fotopoulos V (2020). Impact of an arbuscular mycorrhizal fungal inoculum and exogenous MeJA on fenugreek secondary metabolite production under water deficit. Environ Exp Bot.

[30] Mohammadifard F, Tarakemeh A, Moghaddam M, Zim M (2022). Bentonite mitigates the adverse effects of Drought stress in Fenugreek (*Trigonella foenum-graecum* L). J Soil Sci Plant Nutr.

[31] Jafari M, Shahsavar AR, Talebi M, Hesami M (2022). Exogenous melatonin protects lime plants from drought stress-induced damage by maintaining cell membrane structure, detoxifying ROS and regulating antioxidant systems. Hortic.

[32] Gao W, Zhang Y, Feng Z, Bai Q, He J, Wang Y (2018). Effects of melatonin on antioxidant capacity in naked oat seedlings under drought stress. Mol.

[33] Roy M, Niu J, Irshad A, Kareem HA, Hassan MU, Xu N, Sui X, Guo Z, Amo A, Wang Q (2021). Exogenous melatonin protects alfalfa (*Medicago sativa* L.) seedlings from drought-induced damage by modulating reactive oxygen species metabolism, mineral balance and photosynthetic efficiency. Plant Stress.

[34] Xia H, Ni Z, Hu R, Lin L, Deng H, Wang J, Tang Y, Sun G, Wang X, Li H (2020). Melatonin alleviates drought stress by a non-enzymatic and enzymatic antioxidative system in kiwifruit seedlings. Int J Mol Sci.

[35] Sharma A, Wang J, Xu D, Tao S, Chong S, Yan D, Li Z, Yuan H, Zheng B (2020). Melatonin regulates the functional components of photosynthesis, antioxidant system, gene expression, and metabolic pathways to induce drought resistance in grafted *Carya cathayensis* plants. Sci Total Environ.

[36] Cui G, Sun F, Gao X, Xie K, Zhang C, Liu S, Xi Y (2018). Proteomic analysis of melatonin-mediated osmotic tolerance by improving energy metabolism and autophagy in wheat (*Triticum aestivum* L). Planta.

[37] Li C, Tan D-X, Liang D, Chang C, Jia D, Ma F (2015). Melatonin mediates the regulation of ABA metabolism, free-radical scavenging, and stomatal behaviour in two *Malus* species under drought stress. J Exp Bot.

[38] Vilela RD, Bezerra B, Froehlich A, Endres L (2017). Antioxidant system is essential to increase drought tolerance of sugarcane. Ann Appl Biol.

[39] Sarker U, Oba S (2018). Catalase, superoxide dismutase and ascorbate-glutathione cycle enzymes confer drought tolerance of *Amaranthus tricolor*. Sci Rep.

[40] Dai L, Li J, Harmens H, Zheng X, Zhang C (2020). Melatonin enhances drought resistance by regulating leaf stomatal behaviour, root growth and catalase activity in two contrasting rapeseed (*Brassica napus* L.) genotypes. Plant Physiol Biochem.

[41] Sadak MS, Abdalla AM, Abd Elhamid EM, Ezzo M (2020). Role of melatonin in improving growth, yield quantity and quality of *Moringa oleifera* L. plant under drought stress. Bull Natl Res Cent.

[42] Campos CN, Ávila RG, de Souza KRD, Azevedo LM, Alves JD (2019). Melatonin reduces oxidative stress and promotes drought tolerance in young *Coffea arabica* L. plants. Agric Water Manag.

[43] Dat J, Vandenabeele S, Vranova E, Van Montagu M, Inzé D, Van Breusegem F (2000). Dual action of the active oxygen species during plant stress responses. Cell Mol Life Sci.

[44] Wedeking R, Mahlein A-K, Steiner U, Oerke E-C, Goldbach HE, Wimmer MA (2016). Osmotic adjustment of young sugar beets (*Beta vulgaris*) under progressive drought stress and subsequent rewatering assessed by metabolite analysis and infrared thermography. Funct Plant Biol.

[45] Dghim F, Abdellaoui R, Boukhris M, Neffati M, Chaieb M (2018). Physiological and biochemical changes in *Periploca Angustifolia* plants under withholding irrigation and rewatering conditions. S Afr J Bot.

[46] Huang B, Chen Y-E, Zhao Y-Q, Ding C-B, Liao J-Q, Hu C, Zhou L-J, Zhang Z-W, Yuan S, Yuan M (2019). Exogenous melatonin alleviates oxidative damages and protects photosystem II in maize seedlings under drought stress. Front Plant Sci.

[47] Zamani Z, Amiri H, Ismaili A (2020). Improving drought stress tolerance in fenugreek (*Trigonella foenum-graecum*) by exogenous melatonin. Plant Biosyst.

[48] Ma D, Sun D, Wang C, Li Y, Guo T (2014). Expression of flavonoid biosynthesis genes and accumulation of flavonoid in wheat leaves in response to drought stress. Plant Physiol Biochem.

[49] Abid M, Ali S, Qi LK, Zahoor R, Tian Z, Jiang D, Snider JL, Dai T (2018). Physiological and biochemical changes during drought and recovery periods at tillering and jointing stages in wheat (*Triticum aestivum* L). Sci Rep.

[50] Chakhchar A, Lamaoui M, Aissam S, Ferradous A, Wahbi S, El Mousadik A, Ibnsouda-Koraichi S, Filali-Maltouf A, El Modafar C (2016). Differential physiological and antioxidative responses to drought stress and recovery among four contrasting *Argania spinosa* ecotypes. J Plant Interact.

[51] Wu X, Yuan J, Luo A, Chen Y, Fan Y (2016). Drought stress and re-watering increase secondary metabolites and enzyme activity in *dendrobium moniliform*. Ind Crops Prod.

[52] Nawaz M, Wang Z (2020). Abscisic acid and glycine betaine mediated tolerance mechanisms under drought stress and recovery in *Axonopus compressus*: a new insight. Sci Rep.

[53] Soni P, Abdin MZ (2017). Water deficit-induced oxidative stress affects artemisinin content and expression of proline metabolic genes in *Artemisia annua* L. FEBS Open Bio.

[54] Lyon D, Castillejo MA, Mehmeti-Tershani V, Staudinger C, Kleemaier C, Wienkoop S (2016). Drought and recovery: independently regulated processes highlighting the importance of protein turnover dynamics and translational regulation in *Medicago truncatula*. Mol Cell Proteom.

[55] Cruz de Carvalho MH (2008). Drought stress and reactive oxygen species: production, scavenging and signaling. Plant Signal Behav.

[56] Hosseini MS, Samsampour D, Zahedi SM, Zamanian K, Rahman MM, Mostofa MG, Tran LSP (2021). Melatonin alleviates drought impact on growth and essential oil yield of lemon verbena by enhancing antioxidant responses, mineral balance, and abscisic acid content. Physiol Plant.

[57] Ceraulo L, Ferrugia M, Tesoriere L, Segreto S, Livrea M, Liveri VT (1999). Interactions of melatonin with membrane models: portioning of melatonin in AOT and lecithin reversed micelles. J Pineal Res.

[58] De Lima VR, Caro MS, Munford ML, Desbat B, Dufourc E, Pasa AA, Creczynski-Pasa TB (2010). Influence of melatonin on the order of phosphatidylcholine‐based membranes. J Pineal Res.

[59] García JJ, López-Pingarrón L, Almeida‐Souza P, Tres A, Escudero P, García‐Gil FA, Tan DX, Reiter RJ, Ramírez JM, Bernal‐Pérez M (2014). Protective effects of melatonin in reducing oxidative stress and in preserving the fluidity of biological membranes: a review. J Pineal Res.

[60] Pingping W, Chubin W, Biyan Z (2017). Drought stress induces flowering and enhances carbohydrate accumulation in *Averrhoa Carambola*. Hortic Plant J.

[61] Kazan K, Lyons R (2016). The link between flowering time and stress tolerance. J Exp Bot.

[62] Chauhan J, Kakralya BL, Singhal RK (2017). Evaluation of morpho-physiological attributes of fenugreek (*Trigonella Foenum Graecum*) genotypes under different water regimes. Int J Plant Sci.

[63] Hosseini B, Dehghani H, Khodadadi M. Evaluation of yield and morphological changes in some Iranian endemic fenugreek ecotypes under non-stress and drought stress conditions. Iran J Hortic Sci. 2018;49(3).

[64] Schmalenbach I, Zhang L, Reymond M, Jiménez-Gómez JM (2014). The relationship between flowering time and growth responses to drought in the Arabidopsis Landsberg *erecta* x Antwerp-1 population. Front Plant Sci.

[65] Shi H, Wei Y, Wang Q, Reiter RJ, He C (2016). Melatonin mediates the stabilization of DELLA proteins to repress the floral transition in *Arabidopsis*. J Pineal Res.

[66] Chen D, Wang S, Cao B, Cao D, Leng G, Li H, Yin L, Shan L, Deng X (2016). Genotypic variation in growth and physiological response to drought stress and re-watering reveals the critical role of recovery in drought adaptation in maize seedlings. Front Plant Sci.

[67] Yadav B, Jogawat A, Rahman MS, Narayan OP (2021). Secondary metabolites in the drought stress tolerance of crop plants: a review. Gene Rep.

[68] Selmar D, Kleinwächter M (2013). Influencing the product quality by deliberately applying drought stress during the cultivation of medicinal plants. Ind Crops Prod.

[69] Ghosaliya B, Mittal G, Shivran A, Sharma S, Saxena S, Jain S. Water stress induced changes in seed quality of Fenugreek (*Trigonella foenum-graecum* L.) Genotypes. Legum Res 1:7.

[70] Puente-Garza CA, Meza-Miranda C, Ochoa-Martínez D, García-Lara S (2017). Effect of in vitro drought stress on phenolic acids, flavonols, saponins, and antioxidant activity in Agave salmiana. Plant Physiol Biochem.

[71] Zhang W, Cao Z, Xie Z, Lang D, Zhou L, Chu Y, Zhao Q, Zhang X, Zhao Y (2017). Effect of water stress on roots biomass and secondary metabolites in the medicinal plant *Stellaria Dichotoma* L. *var. lanceolata* Bge. Sci Hortic.

[72] Liao P, Liu D, Xu T-R, Yang Y, Cui X (2017). Soil water stress attenuate the growth and development but enhance the saponin synthesis of *Panax notogesing* during flowering stage. Ind Crops Prod.

[73] Solíz-Guerrero JB, de Rodriguez DJ, Rodríguez-García R, Angulo-Sánchez JL, Méndez-Padilla G: Quinoa saponins: concentration and composition analysis. In: Intrnational Journal of Janick and A Whipkey, editors. Trends in new crops and new uses. Alexandria: ASHS Press. 2002. p. 110–114.

[74] Bidabadi SS, VanderWeide J, Sabbatini P (2020). Exogenous melatonin improves glutathione content, redox state and increases essential oil production in two *Salvia* species under drought stress. Sci Rep.

[75] Naghizadeh M, Kabiri R, Hatami A, Oloumi H, Nasibi F, Tahmasei Z (2019). Exogenous application of melatonin mitigates the adverse effects of drought stress on morpho-physiological traits and secondary metabolites in Moldavian balm (*Dracocephalum moldavica*). Physiol Mol Biol Plants.

[76] Diarra ST, He J, Wang J, Li J (2013). Ethylene treatment improves diosgenin accumulation in in vitro cultures of *Dioscorea Zingiberensis* via up-regulation of CAS and HMGR gene expression. Electron J Biotechnol.

[77] Mohammadi M, Mashayekh T, Rashidi-Monfared S, Ebrahimi A, Abedini D (2020). New insights into diosgenin biosynthesis pathway and its regulation in *Trigonella foenum‐graecum* L. Phytochem Anal.

[78] Mohamadi M, Ebrahimi A, Amerian M (2021). The expression enhancement of some genes involved in th diosgenin biosynthesis pathway in fenugreek treated with different levels of melatonin under salinity stress. Iran J Field Crops Res.

[79] Ciura J, Szeliga M, Grzesik M, Tyrka M (2017). Next-generation sequencing of representational difference analysis products for identification of genes involved in diosgenin biosynthesis in fenugreek (*Trigonella foenum-graecum*). Planta.

[80] Nasrollahi V, Mirzaie-Asl A, Piri K, Nazeri S, Mehrabi R (2014). The effect of drought stress on the expression of key genes involved in the biosynthesis of triterpenoid saponins in liquorice (*Glycyrrhiza glabra*). Phytochem.

[81] Chakma R, Biswas A, Saekong P, Ullah H, Datta A (2021). Foliar application and seed priming of salicylic acid affect growth, fruit yield, and quality of grape tomato under drought stress. Sci Hortic.

[82] Tiwari S, Lata C, Chauhan PS, Nautiyal CS (2016). Pseudomonas putida attunes morphophysiological, biochemical and molecular responses in *Cicer arietinum* L. during drought stress and recovery. Plant Physiol Biochem.

[83] Grintzalis K, Georgiou CD, Schneider Y-J (2015). An accurate and sensitive Coomassie Brilliant Blue G-250-based assay for protein determination. Anal Biochem.

[84] Murshed R, Lopez-Lauri F, Sallanon H (2008). Microplate quantification of enzymes of the plant ascorbate–glutathione cycle. Anal Biochem.

[85] Sales CR, Ribeiro RV, Silveira JA, Machado EC, Martins MO, Lagôa AMM (2013). Superoxide dismutase and ascorbate peroxidase improve the recovery of photosynthesis in sugarcane plants subjected to water deficit and low substrate temperature. Plant Physiol Biochem.

[86] Andrade ER, Ribeiro VN, Azevedo CV, Chiorato AF, Williams TC, Carbonell SA (2016). Biochemical indicators of drought tolerance in the common bean (*Phaseolus vulgaris* L). Euphytica.

[87] Masuko T, Minami A, Iwasaki N, Majima T, Nishimura S-I, Lee YC (2005). Carbohydrate analysis by a phenol–sulfuric acid method in microplate format. Anal Biochem.

[88] Magné C, Larher F (1992). High sugar content of extracts interferes with colorimetric determination of amino acids and free proline. Anal Biochem.

[89] Carillo P, Gibon Y (2011). Protocol: extraction and determination of proline. PrometheusWiki.

[90] Ainsworth EA, Gillespie KM (2007). Estimation of total phenolic content and other oxidation substrates in plant tissues using Folin–ciocalteu reagent. Nat Protoc.

[91] Sembiring EN, Elya B, Sauriasari R. Phytochemical screening, total flavonoid and total phenolic content and antioxidant activity of different parts of Caesalpinia bonduc (L.) Roxb. Phcog Commn. 2018;10(1).

[92] Junglee S, Urban L, Sallanon H, Lopez-Lauri F (2014). Optimized assay for hydrogen peroxide determination in plant tissue using potassium iodide. Am J Anal Chem.

[93] Hodges DM, DeLong JM, Forney CF, Prange RK (1999). Improving the thiobarbituric acid-reactive-substances assay for estimating lipid peroxidation in plant tissues containing anthocyanin and other interfering compounds. Planta.

[94] Del Hierro JN, Herrera T, García-Risco MR, Fornari T, Reglero G, Martin D (2018). Ultrasound-assisted extraction and bioaccessibility of saponins from edible seeds: quinoa, lentil, fenugreek, soybean and lupin. Food Res Int.

[95] Herrera T, Navarro del Hierro J, Fornari T, Reglero G, Martin D (2019). Acid hydrolysis of saponin-rich extracts of quinoa, lentil, fenugreek and soybean to yield sapogenin‐rich extracts and other bioactive compounds. J Sci Food Agric.

