# Supplementary material for: Differential responses of two fenugreek (Trigonella foenum-graecum L.) landraces pretreated with melatonin to prolonged drought stress and subsequent recovery
Source: BMC Plant Biol. 2024 Mar 2;24:161. doi: 10.1186/s12870-024-04835-w (PMC10908034; doi:10.1186/s12870-024-04835-w)
Supplement: Supplementary file 1 — Supplementary Material 1. [file 12870_2024_4835_MOESM1_ESM.docx]

| 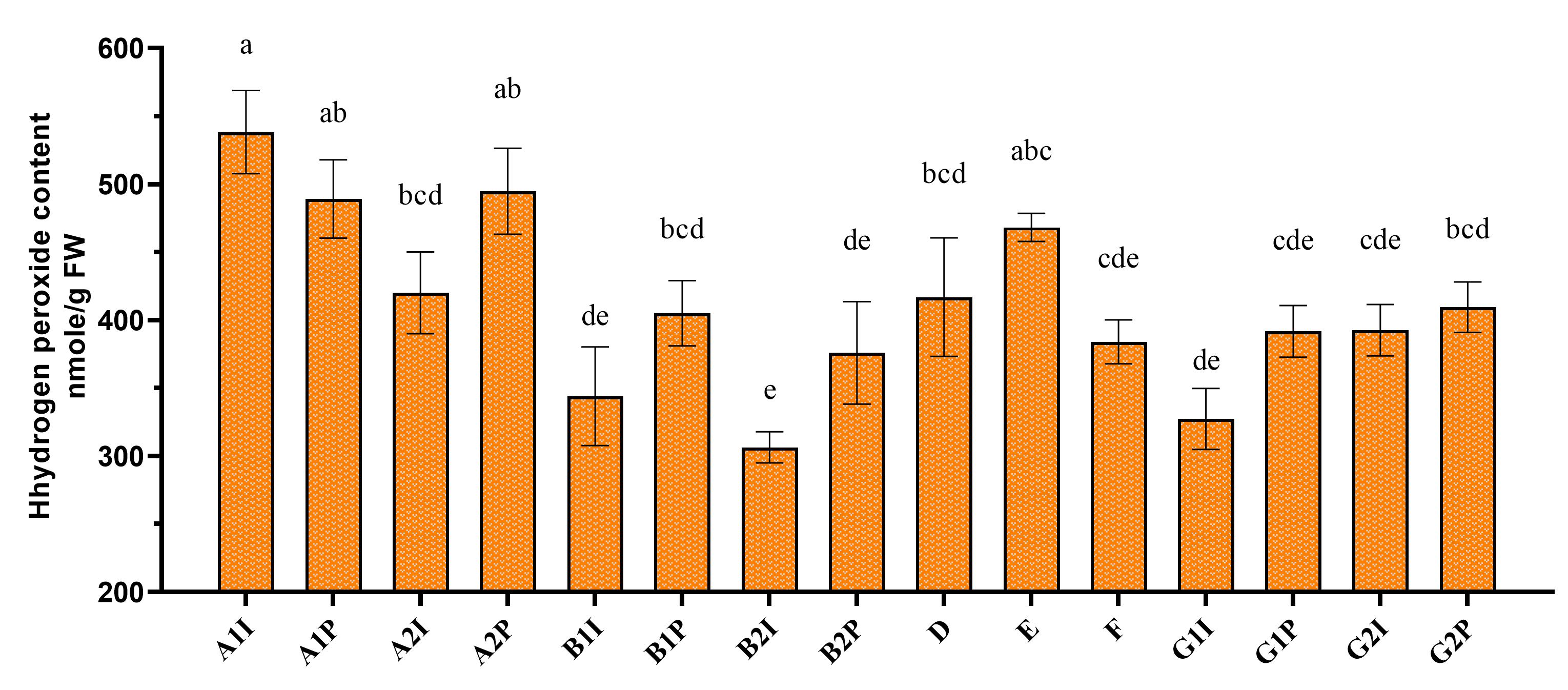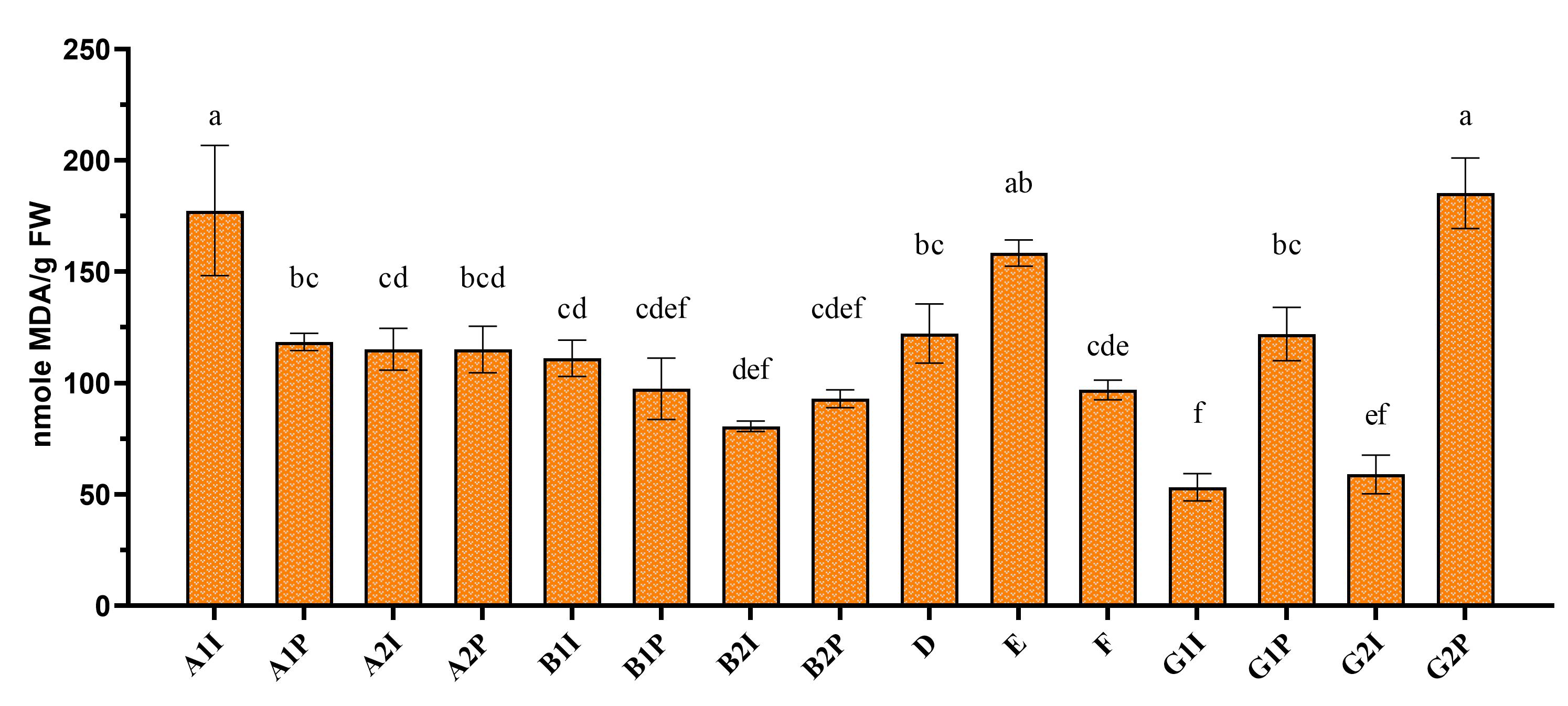 |
| --- |
| Figure S1 The effect of different methods and concentrations of melatonin pretreatment on membrane stability and production of reactive species on fenugreek during drought stress. A: Once melatonin treatment just before the onset of drought stress, B: Twice melatonin treatment with an interval of one week before the beginning of drought stress, D: Drought stress without melatonin treatment, E: mild drought stress was applied before the main drought stress, F: Well-watered plants without melatonin treatment, G: Well-watered plants that were treated twice by melatonin, with an interval of one week before the beginning of drought stress, 1: 100 μM, 2: 20 μM, I: irrigated, P: sprayed. |

| 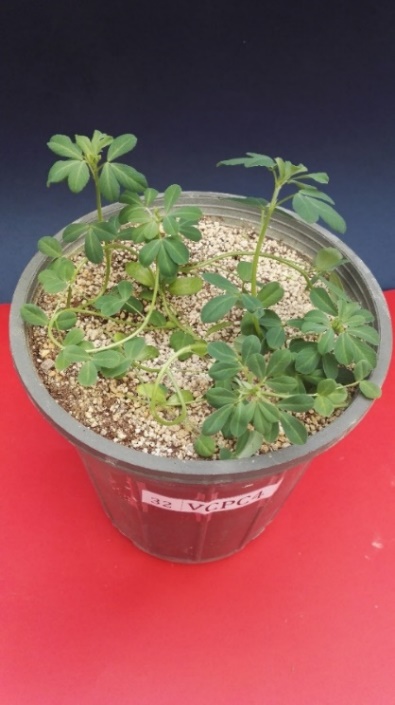 | 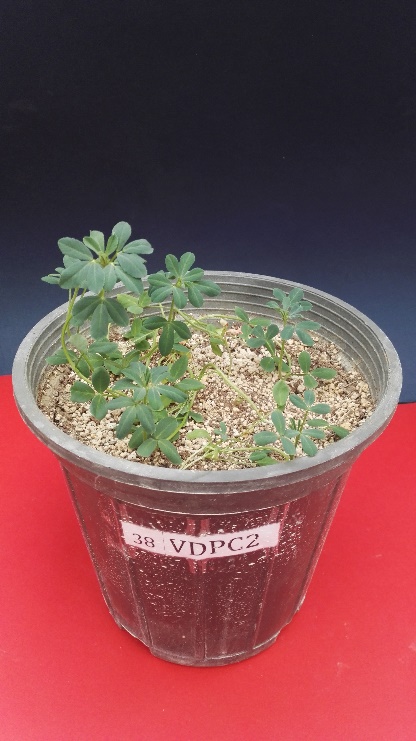 | 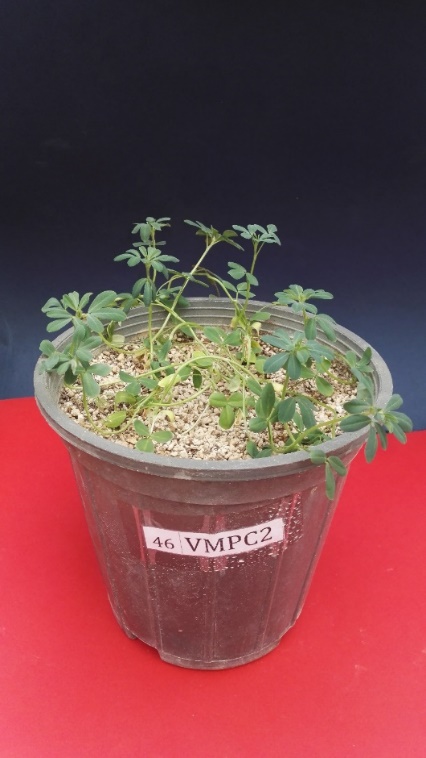 |
| --- | --- | --- |
| **Varamin Control Plants** | **Varamin Drought-stressed Plants** | **Varamin Melatonin-treated Plants** |
| 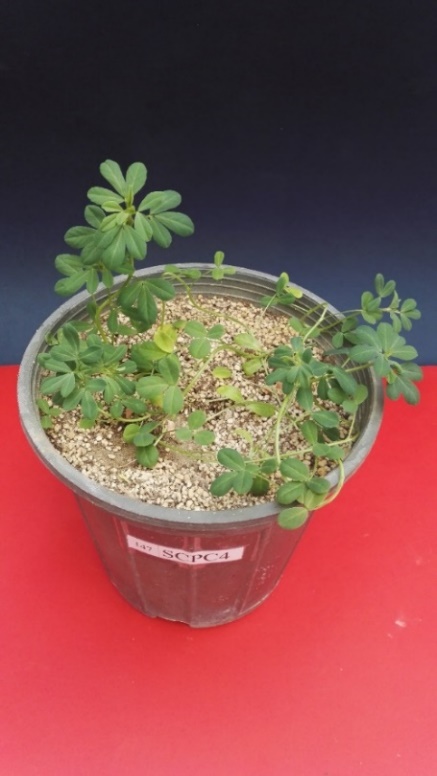 | 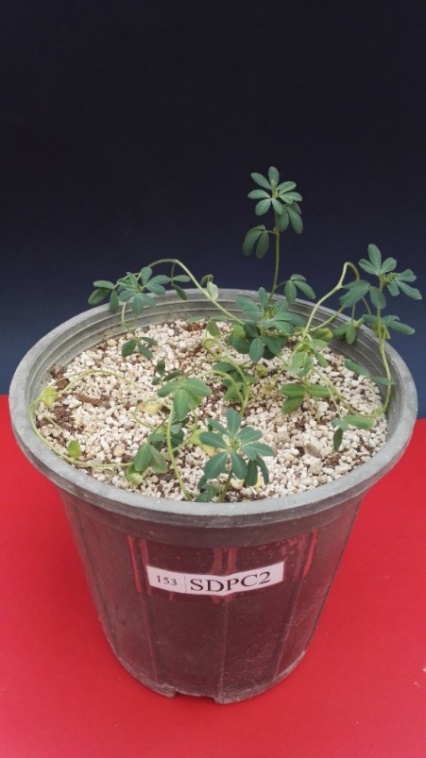 | 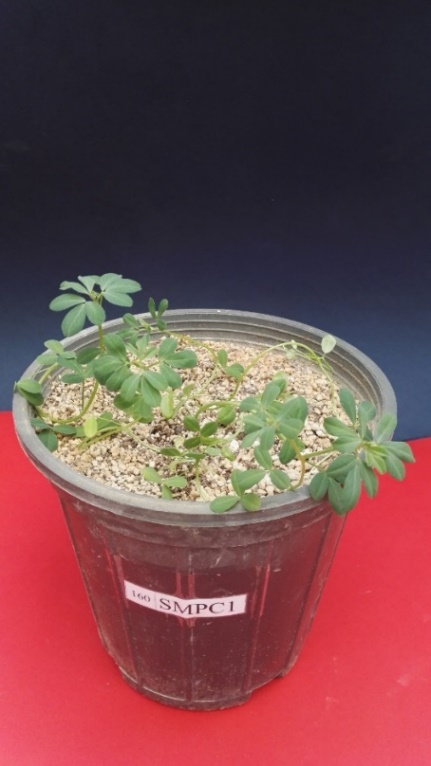 |
| **Shushtar Control Plants** | **Shushtar Drought-stressed Plants** | **Shushtar Melatonin-treated Plants** |
| Figure S2 Plants of Varamin and Shushtar landraces under different treatments at the end of severe drought stress stage. | | |

| Table S1 Primers used for gene expression in the present study. | |
| --- | --- |
| Sequences (5'-3') | Primer Name |
| GGCTCAACCATGATTCTCATACTG | SQS. _Forward_ |
| TACCCACTGTTCCATTGCTATCC | SQS. _Reverse_ |
| AAGAGAGATCCAACACCACTGC | CAS. _Forward_ |
| TACATGACGACGGTATTCTCCC | CAS. _Reverse_ |
| CCTGGTCCTGAGAGCATAACAAATG | BG. Forward |
| CATCAGCCACACCTTGTCCTTC | BG. Reverse |
| TATGTTTGTTGTTGGTGTCAACGAGCAACGAATACAAG | GAPDH. _Forward_ |
| ATGTTAAATGATGCAGCCCTTCCACCTCTC | GAPDH. _Reverse_ |
